# Nuclear envelope transmembrane proteins (NETs) that are up-regulated during myogenesis

**DOI:** 10.1186/1471-2121-7-38

**Published:** 2006-10-24

**Authors:** I-Hsiung Brandon Chen, Michael Huber, Tinglu Guan, Anja Bubeck, Larry Gerace

**Affiliations:** 1 Department of Cell Biology, The Scripps Research Institute, 10555 N. Torrey Pines Rd., La Jolla CA 92037, USA

## Abstract

**Background:**

The nuclear lamina is a protein meshwork lining the inner nuclear membrane, which contains a polymer of nuclear lamins associated with transmembrane proteins of the inner nuclear membrane. The lamina is involved in nuclear structure, gene expression, and association of the cytoplasmic cytoskeleton with the nucleus. We previously identified a group of 67 novel putative nuclear envelope transmembrane proteins (NETs) in a large-scale proteomics analysis. Because mutations in lamina proteins have been linked to several human diseases affecting skeletal muscle, we examined NET expression during differentiation of C2C12 myoblasts. Our goal was to identify new nuclear envelope and lamina components whose expression is coordinated with muscle differentiation.

**Results:**

Using transcriptional microarray analysis, we found that expression of 6 of the NETs significantly increases during myoblast differentiation. We confirmed these results using quantitative RT-PCR, and furthermore, found that all 6 NETs are expressed at high levels in adult mouse skeletal muscle relative to 9 other tissues examined. Using epitope-tagged cDNAs, we determined that the 5 NETs we could analyze (NETs 9, 25, 32, 37 and 39) all target to the nuclear envelope in C2C12 cells. Furthermore, the 3 NETs that we could analyze by immunoblotting were highly enriched in nuclear envelopes relative to microsomal membranes purified from mouse liver. Database searches showed that 4 of the 6 up-regulated NETs contain regions of homology to proteins previously linked to signaling.

**Conclusion:**

This work identified 6 NETs that are predicted to have important functions in muscle development and/or maintenance from their expression patterns during myoblast differentiation and in mouse tissues. We confirmed that 5 of these NETs are authentic nuclear envelope proteins. Four members of this group have potential signaling functions at the NE, based on their sequence homologies.

## Background

The nuclear envelope (NE), which forms the boundary of the nucleus in eukaryotic cells, compartmentalizes nuclear metabolism and helps to organize nuclear structure (reviewed in refs. [1-3]). It contains an inner (INM) and outer nuclear membrane (ONM) separated by the perinuclear lumenal space and joined at nuclear pore complexes (NPCs), giant supramolecular assemblies that mediate molecular trafficking between the nucleus and the cytosol (reviewed in refs. [4,5]). The ONM is continuous with the peripheral ER and in large part, is functionally similar to the latter. In contrast, INM is lined by the nuclear lamina, a meshwork containing a polymer of the lamin intermediate filament proteins as well as other more minor polypeptides, including transmembrane proteins concentrated at the INM (reviewed in refs. [6,7]). Mammals contain 4 major lamin subtypes: lamins A and C, which are encoded by alternatively spliced transcripts of the same gene, and lamins B1 and B2, which are products of separate genes (reviewed in refs. [6-9]). Lamins B1 and B2 are expressed in most somatic cells throughout development, whereas lamins A/C usually are expressed only at or following differentiation.

Genetic and cell biological studies indicate that the nuclear lamina is a structural scaffold that provides mechanical strength to the nucleus and helps to maintain nuclear shape. The lamina also is involved in tethering chromatin and the cytoplasmic cytoskeleton to the NE (reviewed in refs. [10,11]), and mounting evidence implicates the lamina in regulation of gene expression [7,12]. The importance of the lamina in cells is emphasized by recent work showing that over 15 inherited human diseases ("laminopathies") are caused by mutations in lamina components (reviewed in refs. [7,12-14]). Many laminopathies target specific tissues, most commonly cells of skeletal muscle and heart, adipose tissue, or bone and connective tissue, although progeroid syndromes arising from mutations that target NE proteins affect many cell types.

Whereas laminopathies most frequently are caused by mutations in the gene for lamins A/C, human disorders also can arise from mutations in certain transmembrane proteins of the INM. These include emerin, which is linked to Emery-Dreifuss muscular dystrophy, LBR, which is associated with Pelger Huet Anomaly and Greenberg skeletal dysplasia, and MAN1, which is linked to osteopoikilosis and melorheostosis. Diseases with the same clinical symptoms can be caused by mutations in either lamins or in INM proteins (e.g. mutations in either lamins A/C or emerin can cause Emery-Dreifuss muscular dystrophy), emphasizing that some of these proteins have closely linked functions [12,13].

A number of transmembrane proteins of the INM have been connected to different facets of chromatin regulation and NE function. For example, the INM protein LBR in mammals binds to HP1 [15], which is involved in heterochromatin formation. Moreover, the mammalian INM proteins LAP2β, emerin, and MAN1 all contain a ~40 amino acid α-helical "LEM domain" [16]. The LEM domain binds BAF, a DNA-binding protein that appears to be involved in chromosome organization and nuclear assembly (reviewed in ref. [7]). Moreover, multiple INM proteins, including LEM domain proteins, have been found to directly bind to transcriptional regulators (reviewed in ref. [7]). Recently, the LEM protein MAN1 has been shown to attenuate TFG-β signaling due to its direct interaction with Smad transcription factors (reviewed in ref. [17]).

The lamina also has been linked to organization of the cytoplasmic cytoskeleton. Pioneering work in *C. elegans *identified NE transmembrane proteins involved in anchoring the actin cytoskeleton to the NE, including the lamin-interacting UNC-84 protein of the INM and the actin-binding ANC-1 protein of the ONM [18]. Homologous proteins have been described in mammalian cells, including Sun1 of the INM (homologous to UNC-84), which interacts in the perinuclear space with an ONM protein, nesprin 2 giant (homologous to ANC-1), which contains an actin-binding domain [19-21]; reviewed in ref. [11].

Conventional cell biological methods have been successful in identifying ~15 transmembrane proteins (together with their splice variants) that are concentrated at the NE. We recently carried out a large-scale analysis of NE isolated from rodent liver using MudPIT proteomics, and identified an additional 67 novel putative nuclear envelope transmembrane proteins (NETs) [22]. We found that a randomly selected set of 8 of these proteins all target to the NE of cultured cells when expressed by transfection as epitope-tagged fusions [22], confirming that they are authentic NETs and suggesting that most members of this group will prove to be NE proteins.

Striated muscle is one of the tissues most commonly affected by laminopathy mutations [12,13]. With the goal of understanding this disease association, we have analyzed expression of the novel NET genes during differentiation of the murine C2C12 skeletal myoblast cell line. Extensive precedent from studies of tissue development predicts that NETs up-regulated during myoblast differentiation will be important for differentiation and/or for muscle cell maintenance. From this analysis, we identified 6 NETs that are significantly up-regulated during myogensis. We found that these NETs also are highly expressed in mouse muscle, and confirmed that 5 of these are authentic NE proteins. Based on their sequence homologies, many of these proteins could have roles in signal transduction at the NE.

## Results

### Changes in NET expression during C2C12 differentiation

**Figure 1:**
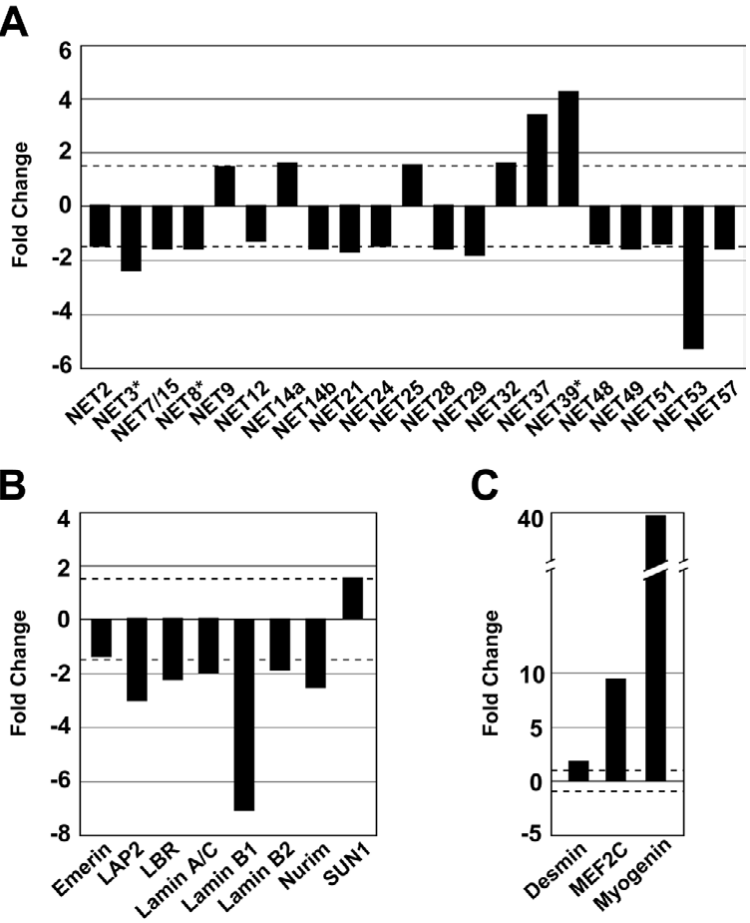
**DNA microarray analysis of proliferating and differentiated C2C12 cells**. Three undifferentiated (baseline) and 3 differentiated (experimental) microarray data sets were obtained from a total of 6 Affymetrix mouse genome 430 2.0 GeneChips™ as described in Materials and Methods. Those genes for which the microarray data had a *p*-value < 0.05 and where the change was ≥~1.5 fold, were deemed to "significantly" change. The NETs that met these two criteria were graphed. (A) up- or down-regulated NETs; (B) NE proteins; (C) muscle differentiation markers. Asterisks indicate previously validated NETs [22]. The dash lines indicate a 1.5 fold change. Abbreviations: Lamin A associated peptide 2 (LAP2) and Myocyte enhancing factor 2C (MEF 2C). Synonym: SUN1 (Unc84a). NET14a is one of the two alternatively spliced transcripts appearing in the new gene annotations, for the originally described NET14.

We have examined the expression of the novel putative/confirmed NETs in mouse C2C12 cells during differentiation, as a means of identifying proteins with a potential role in muscle development. C2C12 cells are an immortalized myoblast line derived from a stem cell ("satellite cell") of adult skeletal muscle involved in muscle regeneration (reviewed in ref. [23]), which expresses the myogenic specification markers MyoD and Myf5. When cultures are shifted to medium containing low serum, the cells withdraw from the cell cycle and ~50–75% of the cell population undergoes differentiation to form multinucleated myotubes [23,24]. The remaining mononucleated cells retain the potential for muscle differentiation upon further culturing [25]. Differentiation of C2C12 cells into muscle involves the induction of "late" myogenic transcriptional regulatory factors including myogenin and MEF2C, followed by expression of the cell cycle inhibitor p21 and muscle-specific structural proteins including components of myofilaments (e.g., myosin heavy chain and troponins). We used Affymetrix DNA microarray analysis to compare the transcript levels of the novel putative/confirmed NETs in proliferating C2C12 cells vs. differentiated populations examined 6 days after shift to low serum medium (Fig. 1). Of the 67 NETs described in our proteomics analysis, 60 could be analyzed with the Affymetrix Mouse Genome 430 v2.0 microarray chip used. Additional File 1 provides an updated description of the original 67 NETs identified in our proteomics analysis, including homology regions identified by BLAST searches of the most recent Pfam, Smart and NCBI COG databases. It also lists the different probe-sets on the Affymetrix 430 v2.0 chip that recognized each of the 60 NET genes detected, and the primers used for analysis of certain transcripts by RT-PCR (described below).

A substantial fraction of the putative/confirmed NETs showed significant changes in their transcript level (i.e., with a *p*-value < 0.05 and showing >~1.5-fold change, dashed lines in Fig. 1) in differentiated C2C12 cultures, as compared to proliferating cells (Fig. 1A). There was a significant increase in the transcript level of 6 of the NETs (NETs 9, 14a, 25, 32, 37 and 39), a significant decrease in the level of 15 of the NETs, and no substantial change for the rest. Many of the well-characterized lamina proteins showed a significant decrease in their transcript level in the differentiated C2C12 cultures, as compared to the proliferating cells (Fig. 1B). Down-regulated transcripts included those for the 3 lamin subtypes and for the transmembrane proteins nurim, LAP2, emerin and LBR. Conversely, there was a significant increase in the transcript level of the transmembrane protein SUN1 (implicated in anchoring of the actin cytoskeleton to the NE; see Background). The levels were not significantly changed for the remaining proteins, including MAN1, LAP1B and LUMA. As expected, the transcript level increased substantially for several markers of terminal myogenic differentiation including desmin (a muscle-specific intermediate filament protein), MEF2C and myogenin (Fig. 1C).

**Figure 2:**
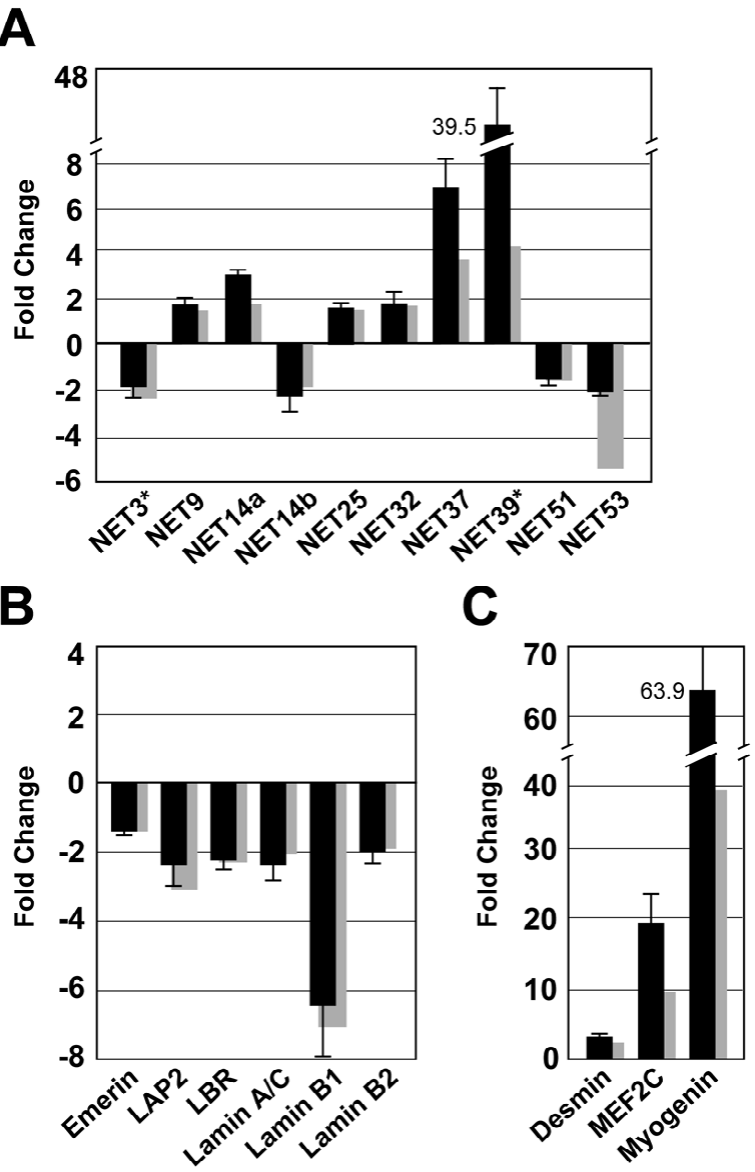
**Validation and extension of microarray analysis by quantitative/real-time PCR. **Primer sets (see Additional File 1) were designed to target the Affymetrix probe set region of the selected genes and to amplify the region across exon-exon junctions whenever possible. Quantitative PCR was performed as described in Materials and Methods. Shown is the mean of all signal values obtained from 6 independent RNA samples. (A) novel NETs; (B) NE markers proteins; (C) muscle differentiation markers. The error bars indicate the standard deviation. Gray columns indicate the fold changes obtained in the microarray analysis, taken from Fig. 1.

To confirm and extend the results of the microarray analysis, we analyzed a number of the transcripts by quantitative real time RT-PCR. This technique provides a more accurate representation of changes in the level of specific transcripts in the differentiated vs. proliferating C2C12 cells than the microarray analysis, since the RT-PCR signal is linear over a broad concentration range [26,27], as validated by our serial dilution studies with various samples (data not shown). We analyzed all 6 of the up-regulated NETs and a sample of 4 of the down-regulated NETs (Fig. 2A). For all of the transcripts examined, the results obtained with the RT-PCR reflected the same up- and down-regulation trends as revealed by the microarray data (Fig. 2A, solid bars, RT-PCR; lightly shaded bars, microarray results taken from Fig. 1; and data not shown), although the magnitude of some of the changes was different. RT-PCR revealed that the levels of NET14a, NET37 and NET39 transcripts increased substantially more than seen by microarray analysis (NET37, 7-fold increase; NET39, 39.5-fold increase; NET14a, 3-fold increase; Fig. 2A), whereas the level of NET53 decreased less by RT-PCR analysis (~2-fold decrease) than seen by microarray. The massive increase in the level of NET39 transcript during C2C12 differentiation indicates a very strong "off/on" control for this gene during terminal myogenesis.

We also examined 4 NETs that showed no significant changes in the microarray analysis, NETs 4, 26, 31 and 56, and found that these transcripts similarly showed no significant changes in RT-PCR (data not shown). As controls, we analyzed transcripts for marker proteins of the NE (Fig. 2B) and of muscle (Fig. 2C). As observed with the NETs, the RT-PCR results were consistent with the microarray data, and some of the transcripts for up-regulated markers (i.e., myogenin and MEF2C) underwent greater increases than observed with the microarray.

**Figure 3:**
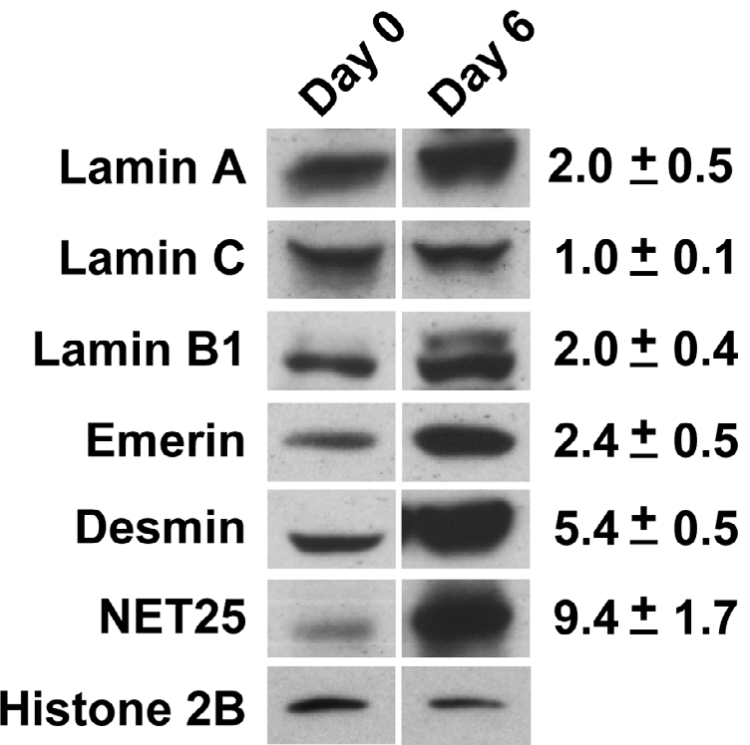
**Immunoblot analysis of marker proteins and NET25 during C2C12 differentiation**. Total proteins from C2C12 cultures harvested either on day 0 (undifferentiated cells) and at day 6 after induction of differentiation were examined by immunoblot analysis and quantification as described in Materials and Methods). Shown are representative images of immunoblots and the calculated fold changes from day 0 to day 6.

**Figure 7:**
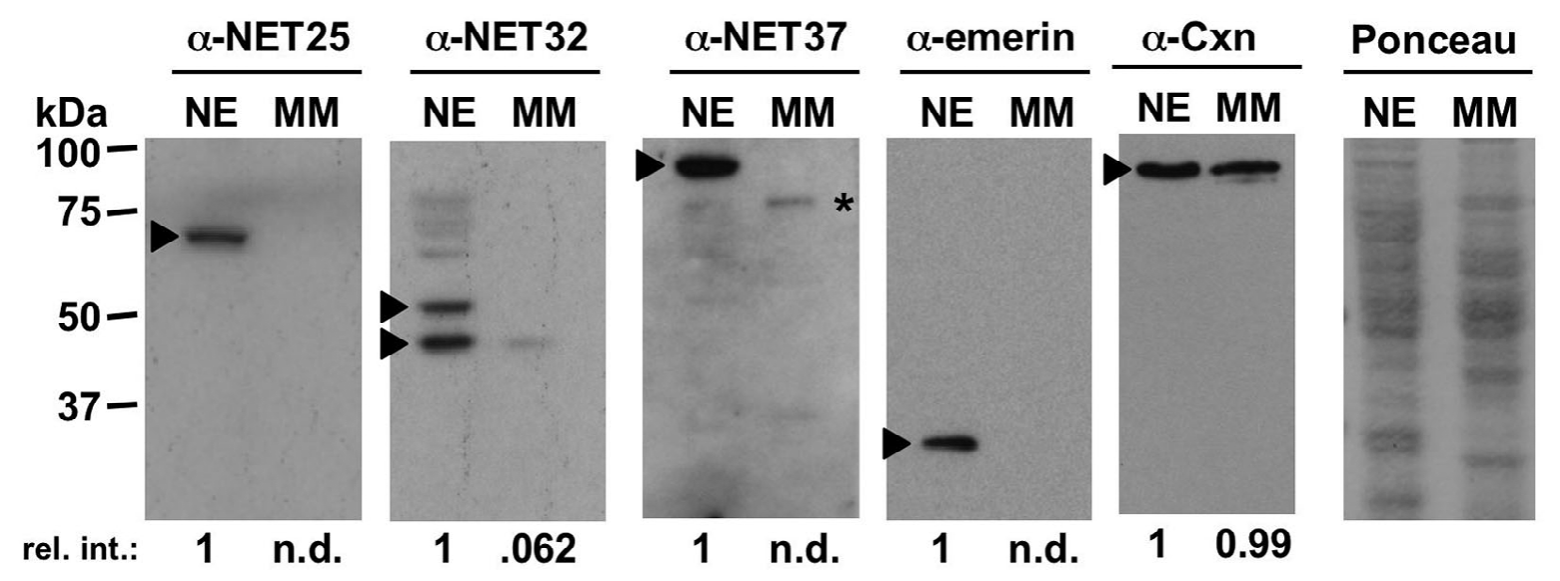
**Relative abundance of NETs in isolated nuclear envelopes and microsomal membranes of liver**. Mouse liver NE and MM fractions were analyzed by immunoblotting with antibodies against NET25, NET32, and NET37 as indicated above each image. Control blots with anti-emerin and anti-calnexin antibodies, as well as a representative Ponceau Red stain of one of the membranes used in these experiments are shown on the right. Positions of the target proteins are indicated by black arrowheads. Labeling of these bands was strongly diminished by pre-incubating the antibodies with the cognate peptides (data not shown), whereas labeling of nonspecific bands was not. A nonspecific in the microsomal lane of the anti-NET37 blot is indicated by an asterisk. Where feasible, signal intensities normalized to the signal in the NE lanes are shown below each membrane.

Since both transcriptional and posttranscriptional mechanisms can regulate gene expression, we decided to examine changes in the protein levels of several NETs and control polypeptides during C2C12 differentiation (Fig. 3). These results were compared to the changes in transcript levels as measured by RT-PCR (Fig. 2). For this analysis we prepared affinity-purified anti-peptide antibodies against NETs 25, 32 and 37, which recognized their cognate antigens in isolated mouse liver NEs by immunoblotting (see Fig. 7 below). However, only 1 of these antibodies (anti-NET25) was sufficiently strong to detect the antigen in whole lysates of C2C12 cells, where the antigens are substantially less enriched than in isolated NEs (Fig. 3). In addition, the antibodies were not useful for immunofluorescence localization. Several well-characterized NE markers (lamins A and C, lamin B1, emerin) and the muscle intermediate filament desmin were analyzed in parallel. We normalized the protein levels to histone H2B, which is proportional to the amount of nuclear DNA and thus to the number of nuclei. H2B levels were closely correlated with the total protein amount in the samples (data not shown).

In all cases examined, we found that the proteins increased to significantly higher levels than predicted by the transcript levels (Fig. 3). Whereas the transcript levels of lamins A/C, lamin B1 and emerin decreased 1.5-6-fold during C2C12 differentiation, the protein levels increased up to 2.4-fold. NET25 protein increased ~9.4-fold (as compared to ~1.5-fold transcript increase), and desmin protein increased ~5.4-fold (as compared to ~3-fold transcript increase. These relative increases in protein amounts, as compared to transcript levels, could result from multiple posttranscriptional mechanisms, including an increase in protein half-life in the differentiated cells, an increase in translation rate, and the cessation of protein dilution by cell growth and division. Irregardless, the positive correlation between increase in transcript and protein levels seen for NET25 and desmin suggest that most or all of the NETs that are transcriptionally up-regulated during C2C12 differentiation will increase at the protein level as well. We predict that the largest increases will occur for NETs14a, 37, and 39, which show the greatest transcript increases.

### NET transcript expression in adult mouse tissues

**Figure 4:**
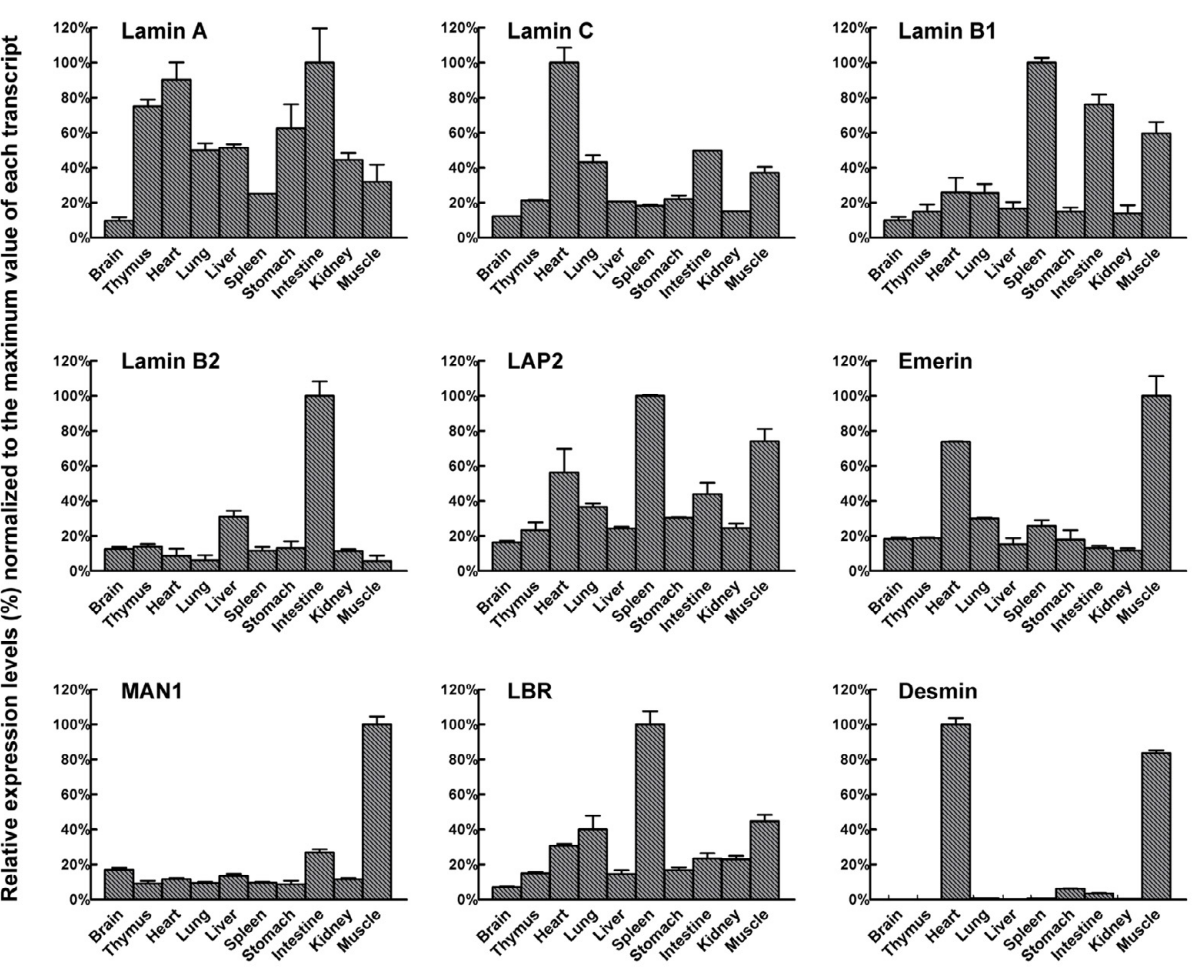
**Expression of NE marker proteins and desmin in 10 tissues of adult mouse**. Transcript levels of selected lamina proteins were measured by quantitative real-time PCR in ten adult C129 mouse organs or tissues. All signal values were normalized to peptidylprolyl isomerase A (Ppia), which was the most consistent reference transcript among four reference transcripts tested across all mouse tissues. The Y-axis indicates the expression level of a gene relative to the highest value (normalized to 100%) in the same tissue. The error bars indicate the standard deviation. The raw data used for preparation of this figure was derived from Additional File 2.

**Figure 5:**
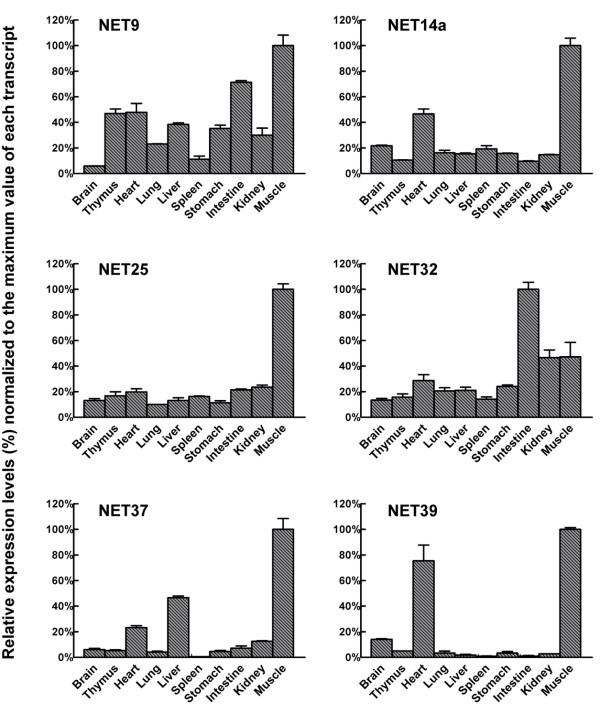
**Expression of NETs up-regulated in C2C12 differentiation in 10 tissues of adult mouse**. Gene expression levels of the up-regulated NETs during C2C12 differentiation were measured by quantitative/real-time PCR in ten adult C129 mouse tissues. All signal values were normalized to peptidylprolyl isomerase A (Ppia) as in Fig. 4. The left Y-axis indicates the expression level of a gene relative to the highest value (normalized to 100%) in the same tissue. The error bars indicate the standard deviation. The raw data used for preparation of this figure was derived from Additional File 3.

To examine whether the 6 NETs whose transcripts are up-regulated during C2C12 myoblast differentiation are expressed highly in skeletal muscle relative to other tissues, we carried out quantitative RT-PCR on 10 adult mouse tissues: brain, thymus, heart, lung, liver, spleen, stomach, intestine and kidney and hindlimb skeletal muscle (Fig. 5). As a benchmark for interpreting these results, we used RT-PCR to compare the transcript levels of desmin and several well-characterized NE markers, including lamins and NE transmembrane proteins, among the 10 mouse tissues (Fig. 4).

Consistent with its known muscle-specific expression, the desmin transcript appeared at a high level in heart and muscle (consisting mainly of striated muscle) and at low levels in stomach and intestine, which contain layers of smooth muscle cells on their outer surfaces. The transcript abundance of the NE markers we tested varied widely among the different tissues. However, many of the markers were expressed at significantly higher levels in 1–3 of the tissues than in the others (i.e., at >2-fold higher levels than in the remaining tissues) (Fig. 4). Selectively high expression was seen for lamin C in heart, lamin B2 in intestine, LBR in spleen, emerin in heart and skeletal muscle, and MAN1 in skeletal muscle.

Our analysis of the mouse tissues revealed that all 6 of the NETs up-regulated in C2C12 differentiation were expressed at relatively high levels in skeletal muscle (Fig. 5). Five of the 6 NETs (NETs 9, 14a, 25, 37 and 39) were expressed at higher levels in skeletal muscle than in any of the other tissues examined, and the 6^th ^(NET32) was expressed at its second highest level (~50% of the maximum) in skeletal muscle. The NETs can be further grouped according to two general types of expression patterns among the set of tissues: (a) NETs 9 and 32 showed relatively high expression (>20% of the maximum level) in the majority of tissues; (b) NETs 14a, 25, 37 and 39 were expressed with strong preference in skeletal muscle (>5-fold higher than in most or all other tissues), although NETs14a, 37, and 39 also were expressed at substantial levels in heart relative to the remaining tissues. The specific cell types where NET expression takes place in the "low expressing" tissues remain to be determined. These conceivably could be relatively minor cell populations (e.g. connective tissue or endothelial cells) in which the expression levels are much higher than the tissue average.

In addition to analyzing adult mouse tissues, we examined the transcript levels for the marker proteins and the 6 NETs at embryonic day 17 (E17) and at postnatal day 7 (P7), as compared to adult (Additional Files 2 and 3). Although organogenesis is essentially completed at E17 in all of the tissues examined except for brain, cell proliferation continues (particularly at P7) and the tissues undergo progressive functional adaptation at the 2 later times. The 6 NETs were expressed at the highest levels at P7 and/or adult, as compared to E17, in almost all of the tissues examined (Additional File 3). A noteworthy exception was NET37, which was expressed at a greater than 10-fold higher level in brain at E17 when neurogenesis is occurring, as compared to the later times. Furthermore, expression of the 6 NETs in heart and skeletal muscle occurred at substantially higher levels in adult tissues (up to 15-fold) than in tissues at E17 and P7. NET39 and NET14a showed the widest range of expression levels among the different tissues examined, indicating that these genes have the most stringent off/on control (Additional File 4). When antibodies of sufficient sensitivity become available, it will be important to directly measure protein levels of NETs across various tissues, since the protein levels may not precisely correspond to the NET transcript levels measured here (see discussion in above section).

### Localization of NETs to the NE

**Figure 6:**
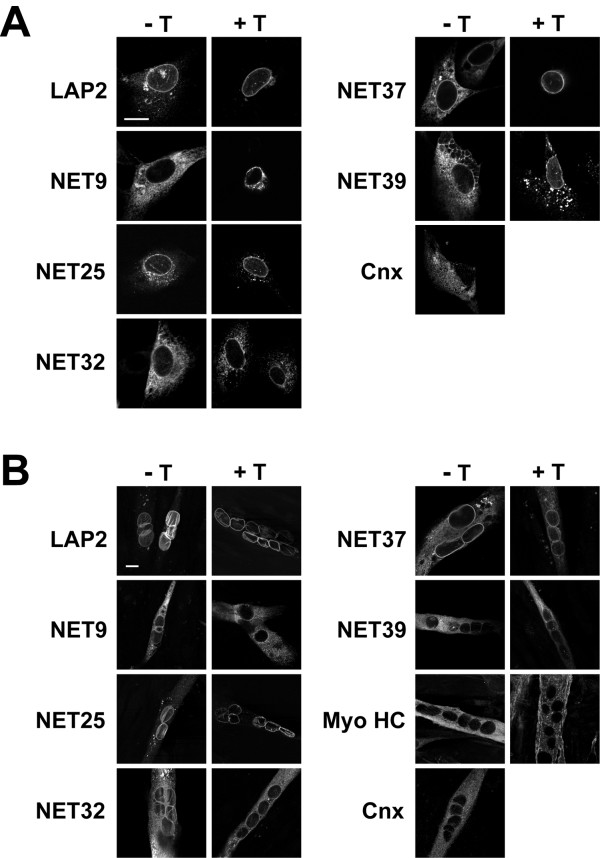
**Nuclear envelope targeting of NETs up-regulated in C2C12 differentiation**. V5 epitope tagged NETs, LAP2β and calnexin (Cnx) were transiently transfected into C2C12 cells. Cell were left untreated (- T) or extracted with 1% Triton X-100 (+ T) before fixation and immunofluorescence staining. Shown are representative images from (A) undifferentiated C2C12 cells, (B) differentiated C2C12 cells. All epitope-tagged calnexin was removed by Triton X-100 pre-extraction (not shown). Scale bar: 20 μm.

We next analyzed whether the 6 NETs that are transcriptionally up-regulated during C2C12 differentiation are *bona fide *NE proteins, as determined by their ability to target to the NE when ectopically expressed. For our analysis, we constructed cDNAs encoding full-length NETs with a V5 epitope tag fused to their C-terminus, and transiently transfected these cDNAs into proliferating C2C12 cells. Cells either were maintained in normal growth medium (Fig. 6A), or were shifted to low serum-containing medium for 6 days after transfection to induce the formation of myotubes (Fig. 6B). They then were fixed and processed for immunofluorescence localization of the transfected proteins, either with (+ T) or without (-T) pre-extraction of cells with buffer containing Triton X-100 before fixation. As seen with the previously characterized NE marker LAP2β (Fig. 6), when NE transmembrane proteins are over-expressed in cultured cells, the proteins become concentrated at the NE but also appear in the peripheral ER, presumably resulting from saturation of NE binding sites (e.g., Ref. [22]. Triton pre-extraction preferentially solubilizes a portion of the peripheral ER pool, whereas the NE form typically remains associated with Triton-insoluble NE substructures (Fig. 6, LAP2β panels). We were able to analyze the expression of all of the NETs except for NET14a, which was not detectable in a significant number of C2C12 cells at any time point after transfection.

In proliferating C2C12 cells, NETs 9, 25, 32, 37 and 39 all targeted to the NE and showed elevated nuclear rim staining relative to adjacent cytoplasmic areas (Fig. 6A). This contrasted with the localization pattern of epitope-tagged calnexin (Cnx), a marker protein for the ER [28], which showed no preferential nuclear rim staining (Fig. 6A). The NE-associated pool of NETs was preferentially retained at the nuclear rim after Triton extraction, whereas a substantial portion of the cytoplasmic pool of the NETs (Fig. 6A), and all of the transfected calnexin (data not shown), was removed. All of the exogenously expressed NETs except for NET39 clearly targeted to the NE in differentiated myotubes (Fig. 6B); however, the cytoplasmic pool of the overexpressed proteins in the myotubes was largely insensitive to extraction by Triton. NET39 expressed in myotubes only occasionally showed elevated nuclear rim labeling relative to the peripheral ER, in contrast to the other NETs. However, NE-localized NET39 in myotubes may be obscured by the substantial cytoplasmic pool that accumulates during the longer expression times used in the myotube experiments. Consistent with our findings with C2C12 myoblasts, we previously found that NET39 targets to the NE of COS7 and HeLa cells in a Triton-resistant form [22]. In C2C12 cells examined without Triton pre-extraction, NETs 9, 32 and 39 typically showed less intense nuclear rim labeling than NETs 25 and 37 (as exemplified by the images presented in Fig. 6), which could reflect lower numbers of binding sites at the NE for the former proteins.

We next used the antibodies we obtained to NETs 25, 32 and 37, which recognize the proteins by immunoblotting, to examine the relative concentration of these proteins in isolated NEs and MMs of mouse liver (Fig. 7). MMs from liver are enriched rough and smooth ER membrane and devoid of NE components [29]. We normalized the signal in the two fractions to the phospholipid content in each sample. We detected specific antigens at the predicted molecular weights for NETs 25, 32 and 37 in the NE fraction, and found that the antigens were highly enriched in NEs as compared to MMs. NETs 25 and 37 were not detectable in MMs under these conditions, similar to emerin (Fig. 7), even when 3-fold more MMs were analyzed by immunoblotting than analyzed in Fig. 7 (data not shown). Based on the lower limit of detection, we estimate that these proteins are >~90-fold enriched in NEs as compared to MMs. On the other hand, NET32 was detectable in MMs, and was 16-fold enriched in NEs as compared to MMs (Fig. 7). In contrast to NET32, the ER resident folding chaperone calnexin had virtually identical concentrations in NEs and MMs. The NE enrichment of these 3 NETs extends the analysis with transfected epitope-tagged proteins, and strongly suggests NE-specific functions for these proteins. Nonetheless the presence of NET32 in the MM fraction, albeit at a substantially lower concentration than in the NE, suggests that it may have additional functions in the peripheral ER.

## Discussion

### Identification and characterization of NETs up-regulated in C2C12 differentiation

We used DNA microarray analysis and quantitative RT-PCR to compare the transcript levels of novel NETs identified in our proteomics analysis [22] in samples from proliferating and differentiated murine C2C12 myoblasts. We found that 6 NETs were significantly up-regulated, and 15 others significantly down-regulated, in differentiated cultures. Based on precedent from studies of tissue development, the up-regulated NETs are likely to have a role in muscle formation and/or maintenance, and NETs that are down-regulated could antagonize these processes.

In agreement with this hypothesis, we found that the 6 up-regulated NET transcripts were expressed highly in skeletal muscle of adult mouse, as compared to 9 other mouse tissues. Of these, NETs 14a, 25, 37 and 39 were expressed with strong preference in skeletal muscle (i.e., >5-fold higher than in most or all other tissues), and NETs 14a, 37 and 39 were also expressed at a substantial level in heart. NETs 9 and 14a showed a broader expression profile, but transcript levels in muscle were still significantly above the average. This confirms and extends our earlier conclusion that NETs have tissue-selective expression patterns [30]. Western blot analysis showed a positive correlation between transcript and protein levels for NET25 and several markers of C2C12 differentiation. While changes in transcript levels are not always reflected in changes of protein levels, this result suggests that other transcriptionally up-regulated NETs probably increase at the protein level.

We found that NETs 9, 25, 32, 37 and 39, when expressed as epitope-tagged proteins in C2C12 myoblasts, were concentrated at the NE in a Triton-resistant manner, indicating that they are authentic NE transmembrane proteins. However, the fraction of ectopically expressed protein that localized to the NE vs. the peripheral ER varied between different NETs as well as between individual cells in a transfected population. Based on the model that accumulation of membrane proteins at the NE is limited mainly by binding [31], this variability may be due to different numbers of binding sites for each NET at the NE, or due to different levels of protein over-expression.

Supporting the results of our NE targeting studies in C2C12 cells, immunoblot analysis of subcellular fractions from mouse liver showed that NETs 25, 32 and 37 were highly enriched in the NE as compared to the MM. NETs 25 and 37 were >~90-fold more concentrated in NEs than in MMs, similar to the NE marker emerin. In contrast, NET32 was only ~16-fold more concentrated in NEs. Since the surface area of the peripheral ER is estimated to be ~100-fold greater than that of the NE in hepatocytes [32], p 661), a major fraction of NET32 apparently resides in the peripheral ER in this cell type. Our results also indicate that localization of proteins to the NE is not an "all or none" phenomenon. Furthermore, the partitioning of different proteins between NE and the peripheral ER may show cell type-specific differences. Thus, NET32 may have functions in both the NE and the peripheral ER, at least in hepatocytes.

### Potential functions of up-regulated NETs

Standard BLAST searches against publicly available databases (Additional File 1) revealed that 4 of the 6 NETs up-regulated in C2C12 differentiation (NETs 25, 32, 37 and 39) contain regions with homology to proteins that have been linked to signal transduction. In addition, the predicted extralumenal region of NET14a contains a WD40 domain, which typically serves as a protein interaction interface [33].

**Figure 8:**
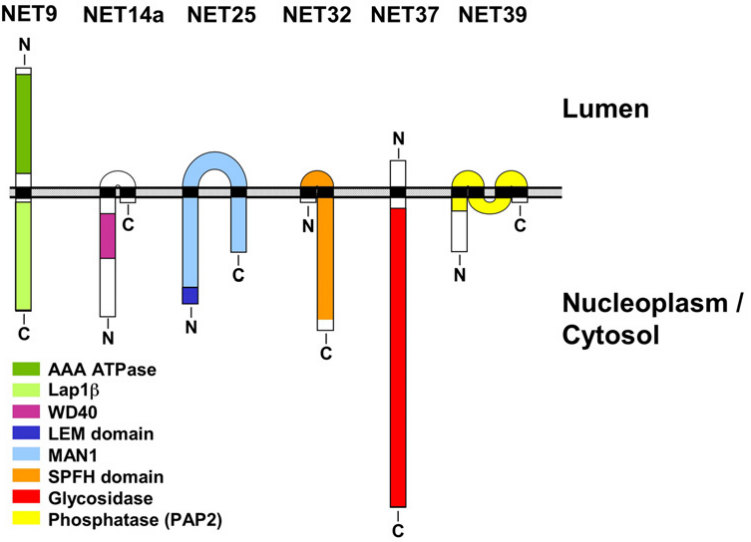
**Model depicting membrane topologies and homologies of NETs up-regulated in C2C12 differentiation**. NET9 (502 residues), NET14a (312 residues), NET25 (511 residues), NET32 (340 residues), NET37 (716 residues) and NET39 (271 residues) are drawn to scale. Regions with homology to known proteins are color coded as indicated. Transmembrane regions, indicated in black, were predicted using the internet based TMPred and TMHMM servers. The phospholipid bilayer is indicated by a hatched rectangle.

A model depicting the predicted membrane topologies and homology domains of the 6 NETs upregulated in C2C12 differentiation is presented in Fig. 8. The proteins are predicted to have 1–4 transmembrane regions and contain one or two sizable extralumenal domains. NET9, also termed LULL1 [34], in addition has a substantial lumenal domain that interacts with torsinA [34]. Whether these proteins reside in the inner or outer nuclear membrane (or both) needs to be resolved by immunoelectron microscopy.

NET25, identified independently as LEM2 [35], shows homology to the first ~770 residues of MAN1 [35], a factor shown to regulate TGF-β signaling (reviewed in ref. [17]). The regions of homology include the N-terminal LEM domain [16], two predicted transmembrane sequences and a winged helix domain that binds DNA [36]. However, NET25 lacks the C-terminal Smad-transcription factor binding domain present in MAN1 [37-40]. Nonetheless, we found that the tissue expression profiles of NET25 and MAN1 are very similar, as both genes have the highest expression in skeletal muscle. Thus, these proteins may have partially overlapping functions.

Almost all of NET32 (Fig. 8), including the two predicted membrane-spanning regions, spans the complete length of the "SPFH" consensus sequence (reviewed in refs. [41,42]). Although the functions of the SPFH domain remain unclear, multiple proteins containing this region occur in sphingolipid-enriched "lipid rafts", which are buoyant membrane derivatives identified by extraction with nonionic detergent. Raft fractions are thought to represent membrane microdomains that mediate signaling events (reviewed in refs. [43-45]). Analogous to functional models proposed for other SPFH domain proteins [41,42], NET32 might be involved in the organization of lipid microdomains at the NE involved in signaling. It is plausible that these hypothetical NE microdomains contain sphingolipids, since there are several reports describing sphingolipids and associated enzymes in the NE (e.g., Refs. [46-48].

The predicted nucleoplasmic/cytosolic domain of NET37 is homologous to family 31 glycosidases (Fig. 8). One potential substrate for the predicted extralumenal glycosidase domain of NET37 is O-linked N-acetylglucosamine (O-linked GlcNAc), which is suggested to have a regulatory role akin to phosphorylation (reviewed in refs. [49,50]). A second potential substrate of NET37 are glycosphingolipids, a fraction of which faces the cytosol in the peripheral ER (reviewed in ref. [51]). Carbohydrate removal from glycosphingolipids generates ceramide, a signaling lipid that plays key roles in differentiation and apoptosis by activating downstream kinases/phosphatases (reviewed in refs. [52,53]). Thus, as either a glycosidase or glycosphingolipid hydrolase, NET37 could play a role in myoblast differentiation.

NET39 has homology to phosphatidic acid phosphatase type 2 in the region that includes its 2 predicted membrane-spanning segments (Fig. 8). If NET39 were a phospholipid phosphatase (reviewed in refs. [54,55]), it could produce the second messenger diacylglycerol, or might attenuate the levels of signaling lipids that enhance cell proliferation, such as sphingosine-1-phosphate or lysophosphatidic acid (reviewed in refs. [53,56]). It is intriguing that the actions of sphingosine-1-phosphate and lysophosphatidic acid (which hypothetically could be degraded by NET39) usually antagonize the actions of ceramide (which hypothetically could be up-regulated by NET37). Thus, the activities of NET37 and NET39, the most highly up-regulated of any of the NETs in C2C12 differentiation, could be coordinately involved in myogenesis by attenuating cell proliferation signals and enhancing differentiation signals. Determining biochemical activities and substrate specificities of NETs 37 and 39 will be key to elucidating these questions.

## Conclusion

We have identified 6 novel NETs with predicted roles in myoblast differentiation and/or muscle maintenance, by analyzing changes in NET expression during C2C12 differentiation, and by comparing expression levels among various adult tissues. Five of these were confirmed to be authentic NE proteins, and 4 have homologies to proteins implicated in signaling. We expect that further analysis of these proteins will shed light on the newly emerging functions of the NE in signal transduction and gene expression.

## Methods

### Plasmid construction

To create expression vectors encoding C-terminally V5-tagged fusion proteins, cDNA sequences were PCR amplified and cloned into pcDNA 3.1 D/V5-His-TOPO (Invitrogen, Carlsbad, CA). NET9, NET32, and NET39 DNA sequences were PCR amplified from the cDNA clones ATCC 10159451, MGC-27952 and MGC-28994 respectively. The primers for PCR amplification of these were

NET9 Fwd: CACCATGTCCCAAAGTCTGAAGAGCCA,

NET9 Rev: CAAAAGGCAGCCTCGCTCTTC,

NET32 Fwd: CACCATGGCTCAGTTGGGAGCTGTTG,

NET32 Rev: GTTCTCCTTTGTGGGTGCCTC,

NET39 Fwd: CACCATGCCAGCTTCCCAGAGCCG, and

NET39 Rev: CCAGGCAGAGATGAGCATCTG. NET14a, NET25, NET37 and calnexin were PCR-amplified from bulk cDNA that we prepared from RNA extracted from C2C12 cells using Transcriptor First Strand cDNA Synthesis Kit™ (Roche Diagnostics, Alameda, CA). The primers for PCR amplification were

NET14a Fwd: CACCATGGCTACAGAAATTGGCTCT,

NET14a Rev: CACAGTAGATAATAAAAAGGAATAACG,

NET25 Fwd: CACCATGGCCGGCCTGTCGGAC,

NET25 Rev: TCGCTCTGAATCAGAGAAAGAGGA,

NET37 Fwd: CACCATGTCCCAGAACCTTCAGGAG,

NET37 Rev: GGACGCCCAGGTAAAGTAGGC,

Cnx Fwd: CACCATGGAAGGGAAGTGGTTACTG, and

Cnx Rev: CTCTCTTCGTGGCTTTCTGTT. NET14b cDNA (WDC146-EGFP) was a kind gift of Dr. Akiko Sakai (Okayama Univ., Okayama, Japan). Rat LAP2β cDNA was previously described [22].

### Antibodies

Anti-peptide antibodies to NETs were prepared by immunization of rabbits with synthetic peptides coupled to pre-activated keyhole limpet hemocyanin (KLH, Imject™, Pierce Biotechnology, Rockford, IL). Each rabbit was injected with a mixture of 2–3 different peptides derived from the same NET, comprising the following sequences (with each peptide containing an N-or C-terminally added cysteine for conjugation to KLH): NET25, residues 49–59 and 63–81; NET32, residues 262–277, 311–325 and 326–340; and NET37, residues 1–15, 18–32 and 322–336. For affinity purification, each peptide was conjugated to CNBr-activated Separose at a concentration of 0.5 mg peptide/ml beads. Typically, antibodies were affinity-purified using a column containing a mixture of the 2–3 peptides used to immunize the animal. Antibodies were eluted from the affinity columns with 4 M MgCl_2_, and were dialyzed against PBS. Polyclonal antibodies to lamins A/C and lamin B1 were prepared in rabbits using the pertinent recombinant proteins, and affinity purified using columns coupled with recombinant proteins. Antibodies to the following antigens were obtained from commercial sources: V5 epitope tag (mouse monoclonal, Invitrogen); desmin and emerin (mouse monoclonal, Novocastra, UK).

### Cell culture

C2C12 myoblasts (ATCC # CRL-1772) were grown in Dulbecco's MEM supplemented with 20% FCS, 100 U/ml penicillin, and 100 μg/ml streptomycin. To induce myoblast differentiation [57], 5 × 10^5 ^C2C12 cells were seeded into a 10 cm, gelatin-coated petri dish. 16 h later, cells were shifted into "low serum" medium consisting of Dulbecco's MEM supplemented with 2% horse serum and antibiotics. This time point was termed "day 0". Multi-nucleated myotubes could be seen by day 3, and increased in number and size by days 5–6. Typically, at least 50% of the nuclei were in multinucleated cells by day 6, the standard time point used for the differentiation experiments.

### Transient transfection of C2C12 cells

C2C12 cells were seeded in 6-well microtiter plates containing gelatin-coated glass coverslips at 1 × 10^5 ^cells/well and allowed to grow for 16 h. Prior to transfection, the growth media containing 20% FCS was removed from cells and replaced with Opti-MEM™ (Invitrogen). cDNA expression plasmids (1μg/well) were transiently transfected into cells using Lipofectamine 2000™ (Invitrogen) at 1:3 ratio (DNA [μg] vs. Lipofectamine [μl]) according to the manufacturer's instructions. The transfection mix was removed from cells 6 hours later and either replaced with the high-serum growth medium to maintain cell proliferation, or with the low-serum media to induce cell differentiation. Proliferating cell cultures were maintained for another 18 to 36 h, whereas differentiated cells cultures were maintained to day 6, and immunofluorescence localization was carried out (below paragraph).

### Immunofluorescence microscopy

Cells were fixed with 4% paraformaldehyde in PBS for 5 minutes at RT, either with or without pre-extraction with Triton X-100 before fixation. For Triton pre-extraction, cells were rinsed once with PBS and then extracted with 1% Triton X-100 in 10 mM Tris-HCl pH 7.5, 1.5 mM MgCl_2 _and 2 mM CaCl_2 _for 30 seconds at RT. Following fixation, cells were treated with 0.5% Triton X-100 in PBS and then incubated for 1 h with a mouse monoclonal antibody against the V5-epitope tag, followed by washing in PBS and incubation for 1h with FITC-conjugated goat-anti-mouse secondary antibody. Chromosomal DNA was stained with TOPRO-3 (Invitrogen, Carlsbad, CA). The stained cells on cover slips were subsequently mounted in Gel Mount™ (Biomeda, Foster City, CA) and sealed with Clarion™ (Biomeda). Cell images were taken with the Bio-Rad/Zeiss MRC1024 laser scanning confocal microscope and analyzed by LaserSharp 2000™ software (Bio-Rad). Images were pseudo-colorized and merged with Adobe Photoshop CS™.

### RNA preparation

To extract RNA from C2C12 cells, proliferating or differentiated cultures grown on 10-cm petri dishes were rinsed once with PBS and directly lysed in 1 ml of Trizol™ (Invitrogen) following the manufacturer's instructions. RNA samples were further treated with TURBO DNase™ (Ambion, Inc., Austin, TX) and purified by RNeasy Total RNA Isolation Kit™ (Qiagen, Valencia, CA). RNA purity was tested by spectrophotometry, and RNA integrity was verified by agarose gel electrophoresis to visualize ribosomal RNA. To limit variance in the microarray experiments, each RNA sample that was analyzed was pooled from 3 separate culture plates that were grown at the same time, and 3 such RNA pools prepared from cultures set up at sequential one-day intervals were further combined. To extract RNA from mouse tissues, the 10 tissues or organs indicated were surgically removed from C129 mice on embryonic day 17 (E17), post-natal day 7 (P7) or ~10 week old mice (adult). Care was taken to remove fat from hindlimb skeletal muscle. Upon removal, tissues were immediately immersed in RNA Later™ (Ambion, Inc., Austin, TX) and stored at -80°C to preserve RNA integrity. Tissues or organs from 3–4 mice were pooled and homogenized in a hand-held homogenizer. RNA samples were extracted and purified as described above.

### DNA microarray analysis

DNA microarray hybridization was carried out by the TSRI DNA microarray core facility [58]. Briefly, 5 μg of total RNA were used to synthesize cDNA that was then used as a template to generate biotinylated cRNA. cRNA was fragmented and hybridized to the Affymetrix GeneChip Mouse Genome 430 2.0™ array (Affymetrix, Santa Clara, CA), which allows analysis of over 39,000 transcripts selected from GenBank, dbEST, and RefSeq. The probe set for each gene on this microarray chip contains 11 perfectly matched probes and 11 control probes with a 1 bp-mismatch; moreover, some genes are represented by multiple probe sets (Additional File 1). The arrays were then washed and scanned with a laser scanner, and images were analyzed by using Affymetrix Microarray SuiteVersion 5.0™ (MAS 5.0; Affymetrix). The raw data of microarray signal values were deposited in Gene Expression Omnibus (GEO accession #GSE4694). Three different RNA samples (see above) from proliferating and from differentiated C2C12 cells were individually analyzed (6 microarrays total). The data were further normalized by GCRMA [59] to eliminate probe level anomaly, and were subjected to a Bayesian regularized *t*-test [60], comparing proliferating cells ("baseline") and differentiated cells ("experimental"), to obtain a global false discovery rate measurement and confidence level assessment, which is indicated as a *p*-value for the significance of expression changes for each probe set. A list of probe sets used to measure NET expression level is provided in Additional File 1. The expression change was calculated by dividing the experimental (differentiated cells) signal values by the baseline (proliferating cells) values for up-regulated genes, and *vice versa *for down-regulated genes. Only data from probe sets with a *p*-value of less than 0.05 in the regularized *t*-test were deemed "significant" and were used to calculate the average expression change of NETs.

### Quantitative real-time PCR

Quantitative real-time PCR was performed using a LightCycler 2.0 thermal cycler system according to the manufacturer's instructions (Roche Diagnostics). RT-PCR primers were designed to target the sequences close to Affymetrix probe regions and the sequences across the exon-exon junction when it was possible (Additional File 1). 2 μg of total RNA was first reverse-transcribed to cDNA using Transcriptor First Strand cDNA Synthesis Kit™ (Roche Diagnostics). The cDNA sample was then used to measure expression level of a gene. The targeted gene was amplified using LightCycler FastStart DNA Master^PLUS ^SYBR Green I™ (Roche Diagnostics) with a Touch-Down PCR protocol, where annealing temperature was ramped down from 68°C to 55°C to target a broad range of probe annealing temperatures. A standard curve that was used to correct different amplification efficiencies was generated for each gene using a dilution series of cDNA prepared from C2C12 total RNA. Two housekeeping genes, Hprt and Ppia, were chosen for "Reference" transcripts in every quantitative PCR run because both genes had consistent expression levels across all tested samples. The "Calibrator", a cDNA sample prepared from C2C12 total RNA in bulk, was also included in each PCR run to correct variances among reagents and thermocycler runs.

### Immunoblot analysis

To quantify levels of NE proteins and desmin at day 0 and day 6 after differentiation, 3 different C2C12 differentiation experiments conducted on separate days were examined. Total cell protein was harvested on day 0 and day 6 after induction of differentiation, and equal amounts of total protein from each sample were loaded on SDS gels. Proteins were separated by electrophoresis, transferred to a nitrocellulose membrane, and subjected to immunoblot analysis. The analysis was performed using primary antibodies against lamins A and C, lamin B1, NET25, emerin, histone H2B and desmin, and horseradish peroxidase-conjugated secondary antibodies. Proteins were detected by SuperSignal West Pico™ (Pierce). X-ray films were scanned on Bio-Rad GS-800 Calibrated Densitometer (Bio-Rad, Hercules, CA). The intensities of the corresponding protein bands were quantified by QuantityOne software (Bio-Rad). Similar methods were used to compare the concentrations of NETs in isolated NEs and MMs.

### Subcellular fractionation of mouse liver

Mouse liver NEs were isolated as described [61], and mouse liver MMs were prepared as before [22]. The analysis in Fig. 7 involved NEs that were pre-extracted with 0.5 M NaCl to remove most chromatin contamination [61].

### Colorimetric determination of phospholipids

Phospholipids in mouse liver NE and microsomal membrane (MM) fractions were determined using a miniaturized method based on the complex formation between phospholipids and ammonium thiocyanate [62]. Briefly, 20 μl of NEs or microsomal membranes (MMs) were extracted with 400 μl chloroform: methanol (2 : 1) and concentrated. Samples were diluted with chloroform to a total volume of 150 μl and mixed with an equal volume of ammonium thiocyanate. The amount of water insoluble complex was determined spectrophotometrically at a wavelength of 460 nm. Phospholipid concentrations were determined using a standard curve produced with L-α-phosphatidylcholine dissolved in chloroform (Sigma, St. Louis, MO).

## Authors' contributions

IBC designed and executed most of the experiments, carried out most of the bioinformatics analysis, and helped to conceptualize the project and interpret the results. MH executed the experiments in Fig. 7, carried out database analysis used for Fig. 8, and helped with the interpretation of the results and the design of the manuscript. TG prepared and characterized the anti-NET antibodies. AB set up the C2C12 myoblast differentiation system and contributed to experimental design. LG coordinated the design of the experiments, led the conceptualization of the project, and drafted the manuscript.

## Supplementary Material

Additional File 1 NETs and control genes examined by microarray and RT-PCR analysis. List of all genes analyzed in this study. This includes an updated list of NETs originally described in Ref. [22] together with gene annotations from standard BLAST analysis, a list of primers used for quantitative RT-PCR, and the probesets on Affymetrix Mouse Genome 430 2.0™ array that were recognized by the specific genes indicated Click here for file

Additional File 2 Relative levels of NE markers and desmin at three developmental stages in 10 mouse tissues. Gene expression levels of selected lamina proteins were measured by quantitative real-time PCR in ten C129 mouse tissues from embryonic day 17 (E7), post-natal day 7 (P7) and adult. All signal values were normalized to peptidylprolyl isomerase A (Ppia) as in Fig. 4. The Y-axis indicates the signal value of RNA transcript after normalization to Ppia. The error bars indicate the standard deviation. Click here for file

Additional File 3 **Relative levels of NETs up-regulated in C2C12 differentiation at three developmental stages in 10 mouse tissues**. Gene expression levels of the 6 NETs up-regulated during C2C12 differentiation were measured by quantitative real-time PCR in ten C129 mouse tissues from embryonic day 17 (E7), post-natal day 7 (P7) and adult. All signal values were normalized to peptidylprolyl isomerase A (Ppia) as in Fig. 4. The Y-axis indicates the signal value of RNA transcript after normalization to Ppia. The error bars indicate the standard deviation. Click here for file

Additional File 4 Expression range of selected lamina proteins and the up-regulated NETs. A box-and-whisker graph shows gene expression levels of selected lamina proteins and the NETs in all tested mouse tissues. The expression values measured by RT-PCR in Additional File 2 and Additional File 3 were plotted on a log base 10 scale. Boxes indicate the median, the lower quartile and upper quartile values. Whiskers indicate the range of data points from the highest to the lowest. See [63]. Click here for file

## References

[B1] Gerace L, Burke B (1988). Functional organization of the nuclear envelope. Annu Rev Cell Biol.

[B2] Holaska JM, Wilson KL, Mansharamani M (2002). The nuclear envelope, lamins and nuclear assembly. Curr Opin Cell Biol.

[B3] Hetzer MW, Walther TC, Mattaj IW (2005). Pushing the envelope: structure, function, and dynamics of the nuclear periphery. Annu Rev Cell Dev Biol.

[B4] Tran EJ, Wente SR (2006). Dynamic nuclear pore complexes: life on the edge. Cell.

[B5] Fahrenkrog B, Koser J, Aebi U (2004). The nuclear pore complex: a jack of all trades?. Trends Biochem Sci.

[B6] Stuurman N, Heins S, Aebi U (1998). Nuclear lamins: their structure, assembly, and interactions. J Struct Biol.

[B7] Gruenbaum Y, Margalit A, Goldman RD, Shumaker DK, Wilson KL (2005). The nuclear lamina comes of age. Nat Rev Mol Cell Biol.

[B8] Hutchison CJ (2002). Lamins: building blocks or regulators of gene expression?. Nat Rev Mol Cell Biol.

[B9] Goldman RD, Gruenbaum Y, Moir RD, Shumaker DK, Spann TP (2002). Nuclear lamins: building blocks of nuclear architecture. Genes Dev.

[B10] Starr DA, Han M (2003). ANChors away: an actin based mechanism of nuclear positioning. J Cell Sci.

[B11] Worman HJ, Gundersen GG (2006). Here come the SUNs: a nucleocytoskeletal missing link. Trends Cell Biol.

[B12] Worman HJ, Courvalin JC (2005). Nuclear envelope, nuclear lamina, and inherited disease. Int Rev Cytol.

[B13] Mounkes L, Stewart CL (2004). Structural organization and functions of the nucleus in development, aging, and disease. Curr Top Dev Biol.

[B14] Hutchison CJ, Worman HJ (2004). A-type lamins: guardians of the soma?. Nat Cell Biol.

[B15] Ye Q, Worman HJ (1996). Interaction between an integral protein of the nuclear envelope inner membrane and human chromodomain proteins homologous to Drosophila HP1. J Biol Chem.

[B16] Lin F, Blake DL, Callebaut I, Skerjanc IS, Holmer L, McBurney MW, Paulin-Levasseur M, Worman HJ (2000). MAN1, an inner nuclear membrane protein that shares the LEM domain with lamina-associated polypeptide 2 and emerin. J Biol Chem.

[B17] Worman HJ (2006). Inner nuclear membrane and regulation of Smad-mediated signaling. Biochim Biophys Acta.

[B18] Starr DA, Fischer JA (2005). KASH 'n Karry: the KASH domain family of cargo-specific cytoskeletal adaptor proteins. Bioessays.

[B19] Crisp M, Liu Q, Roux K, Rattner JB, Shanahan C, Burke B, Stahl PD, Hodzic D (2006). Coupling of the nucleus and cytoplasm: role of the LINC complex. J Cell Biol.

[B20] Padmakumar VC, Libotte T, Lu W, Zaim H, Abraham S, Noegel AA, Gotzmann J, Foisner R, Karakesisoglou I (2005). The inner nuclear membrane protein Sun1 mediates the anchorage of Nesprin-2 to the nuclear envelope. J Cell Sci.

[B21] Haque F, Lloyd DJ, Smallwood DT, Dent CL, Shanahan CM, Fry AM, Trembath RC, Shackleton S (2006). SUN1 interacts with nuclear lamin A and cytoplasmic nesprins to provide a physical connection between the nuclear lamina and the cytoskeleton. Mol Cell Biol.

[B22] Schirmer EC, Florens L, Guan T, Yates JR, Gerace L (2003). Nuclear membrane proteins with potential disease links found by subtractive proteomics. Science.

[B23] Charge SB, Rudnicki MA (2004). Cellular and molecular regulation of muscle regeneration. Physiol Rev.

[B24] Bains W, Ponte P, Blau H, Kedes L (1984). Cardiac actin is the major actin gene product in skeletal muscle cell differentiation in vitro. Mol Cell Biol.

[B25] Yoshida N, Yoshida S, Koishi K, Masuda K, Nabeshima Y (1998). Cell heterogeneity upon myogenic differentiation: down-regulation of MyoD and Myf-5 generates 'reserve cells'. J Cell Sci.

[B26] Kuo WP, Liu F, Trimarchi J, Punzo C, Lombardi M, Sarang J, Whipple ME, Maysuria M, Serikawa K, Lee SY, McCrann D, Kang J, Shearstone JR, Burke J, Park DJ, Wang X, Rector TL, Ricciardi-Castagnoli P, Perrin S, Choi S, Bumgarner R, Kim JH, Short GF, Freeman MW, Seed B, Jensen R, Church GM, Hovig E, Cepko CL, Park P, Ohno-Machado L, Jenssen TK (2006). A sequence-oriented comparison of gene expression measurements across different hybridization-based technologies. Nat Biotechnol.

[B27] Qin LX, Beyer RP, Hudson FN, Linford NJ, Morris DE, Kerr KF (2006). Evaluation of methods for oligonucleotide array data via quantitative real-time PCR. BMC Bioinformatics.

[B28] Kleizen B, Braakman I (2004). Protein folding and quality control in the endoplasmic reticulum. Curr Opin Cell Biol.

[B29] Dallner G (1974). Isolation of rough and smooth microsomes--general. Methods Enzymol.

[B30] Schirmer EC, Florens L, Guan T, Yates JR, Gerace L (2005). Identification of novel integral membrane proteins of the nuclear envelope with potential disease links using subtractive proteomics. Novartis Found Symp.

[B31] Holmer L, Worman HJ (2001). Inner nuclear membrane proteins: functions and targeting. Cell Mol Life Sci.

[B32] Alberts B, Johnson A, Lewis J, Raff M, Roberts K, Walter P (2002). Molecular Biology of the Cell.

[B33] Neer EJ, Smith TF (2000). A groovy new structure. Proc Natl Acad Sci U S A.

[B34] Goodchild RE, Dauer WT (2005). The AAA+ protein torsinA interacts with a conserved domain present in LAP1 and a novel ER protein. J Cell Biol.

[B35] Brachner A, Reipert S, Foisner R, Gotzmann J (2005). LEM2 is a novel MAN1-related inner nuclear membrane protein associated with A-type lamins. J Cell Sci.

[B36] Caputo S, Couprie J, Duband-Goulet I, Konde E, Lin F, Braud S, Gondry M, Gilquin B, Worman HJ, Zinn-Justin S (2006). The Carboxyl-terminal Nucleoplasmic Region of MAN1 Exhibits a DNA Binding Winged Helix Domain. J Biol Chem.

[B37] Raju GP, Dimova N, Klein PS, Huang HC (2003). SANE, a novel LEM domain protein, regulates bone morphogenetic protein signaling through interaction with Smad1. J Biol Chem.

[B38] Osada S, Ohmori SY, Taira M (2003). XMAN1, an inner nuclear membrane protein, antagonizes BMP signaling by interacting with Smad1 in Xenopus embryos. Development.

[B39] Lin F, Morrison JM, Wu W, Worman HJ (2005). MAN1, an integral protein of the inner nuclear membrane, binds Smad2 and Smad3 and antagonizes transforming growth factor-beta signaling. Hum Mol Genet.

[B40] Pan D, Estevez-Salmeron LD, Stroschein SL, Zhu X, He J, Zhou S, Luo K (2005). The integral inner nuclear membrane protein MAN1 physically interacts with the R-Smad proteins to repress signaling by the transforming growth factor-{beta} superfamily of cytokines. J Biol Chem.

[B41] Langhorst MF, Reuter A, Stuermer CA (2005). Scaffolding microdomains and beyond: the function of reggie/flotillin proteins. Cell Mol Life Sci.

[B42] Morrow IC, Parton RG (2005). Flotillins and the PHB domain protein family: rafts, worms and anaesthetics. Traffic.

[B43] Schuck S, Simons K (2004). Polarized sorting in epithelial cells: raft clustering and the biogenesis of the apical membrane. J Cell Sci.

[B44] Parton RG, Richards AA (2003). Lipid rafts and caveolae as portals for endocytosis: new insights and common mechanisms. Traffic.

[B45] Kusumi A, Suzuki K (2005). Toward understanding the dynamics of membrane-raft-based molecular interactions. Biochim Biophys Acta.

[B46] Wu G, Lu ZH, Ledeen RW (1995). Induced and spontaneous neuritogenesis are associated with enhanced expression of ganglioside GM1 in the nuclear membrane. J Neurosci.

[B47] Vielhaber G, Pfeiffer S, Brade L, Lindner B, Goldmann T, Vollmer E, Hintze U, Wittern KP, Wepf R (2001). Localization of ceramide and glucosylceramide in human epidermis by immunogold electron microscopy. J Invest Dermatol.

[B48] Alessenko A, Chatterjee S (1995). Neutral sphingomyelinase: localization in rat liver nuclei and involvement in regeneration/proliferation. Mol Cell Biochem.

[B49] Love DC, Hanover JA (2005). The hexosamine signaling pathway: deciphering the "O-GlcNAc code". Sci STKE.

[B50] Zachara NE, Hart GW (2004). O-GlcNAc a sensor of cellular state: the role of nucleocytoplasmic glycosylation in modulating cellular function in response to nutrition and stress. Biochim Biophys Acta.

[B51] Tettamanti G (2004). Ganglioside/glycosphingolipid turnover: new concepts. Glycoconj J.

[B52] Hannun YA, Obeid LM (2002). The Ceramide-centric universe of lipid-mediated cell regulation: stress encounters of the lipid kind. J Biol Chem.

[B53] Ogretmen B, Hannun YA (2004). Biologically active sphingolipids in cancer pathogenesis and treatment. Nat Rev Cancer.

[B54] Sigal YJ, McDermott MI, Morris AJ (2005). Integral membrane lipid phosphatases/phosphotransferases: common structure and diverse functions. Biochem J.

[B55] Pyne S, Long JS, Ktistakis NT, Pyne NJ (2005). Lipid phosphate phosphatases and lipid phosphate signalling. Biochem Soc Trans.

[B56] Mills GB, Moolenaar WH (2003). The emerging role of lysophosphatidic acid in cancer. Nat Rev Cancer.

[B57] Silberstein L, Webster SG, Travis M, Blau HM (1986). Developmental progression of myosin gene expression in cultured muscle cells. Cell.

[B58] http://www.scripps.edu/researchservices/dna_array/.

[B59] Wu Z, Irizarry RA (2005). Stochastic models inspired by hybridization theory for short oligonucleotide arrays. J Comput Biol.

[B60] Baldi P, Long AD (2001). A Bayesian framework for the analysis of microarray expression data: regularized t -test and statistical inferences of gene changes. Bioinformatics.

[B61] Snow CM, Senior A, Gerace L (1987). Monoclonal antibodies identify a group of nuclear pore complex glycoproteins. J Cell Biol.

[B62] Stewart JC (1980). Colorimetric determination of phospholipids with ammonium ferrothiocyanate. Anal Biochem.

[B63] http://ellerbruch.nmu.edu/cs255/jnord/boxplot.

