# Supplementary material for: Nuclear envelope transmembrane proteins (NETs) that are up-regulated during myogenesis
Source: BMC Cell Biol. 2006 Oct 24;7:38. doi: 10.1186/1471-2121-7-38 (PMC1635557; doi:10.1186/1471-2121-7-38)
Supplement: Additional File 1 — NETs and control genes examined by microarray and RT-PCR analysis. List of all genes analyzed in this study. This includes an updated list of NETs originally described in Ref. [22] together with gene annotations from standard BLAST analysis, a list of primers used for quantitative RT-PCR, and the probesets on Affymetrix Mouse Genome 430 2.0™ array that were recognized by the specific genes indicated [file 1471-2121-7-38-S1.doc]

|  | **Gene Name** | **Accession Number**  **[Mus musculus]** | **Conserved**  **Protein Domain(s) (7)** | **RT-PCR Primers** | **Affymetrix Probeset(s)** |
| --- | --- | --- | --- | --- | --- |
| Desmin | Des | >gi|33563250|ref|NP_034173.1| desmin |  | Fwd:CAGCCTCTTCCACTCTCG  Rev:GCTTTGACACCTGCCAATAG | 1426731_at  1426732_at |
| Emerin | Emd | >gi|6679641|ref|NP_031953.1| emerin |  | Fwd:GCCTTTCTGCTCTTTGTTTACTAT  Rev:CCTTAGGCTAGACCTTGCAC | 1417357_at |
| Hmbs | Hmbs | >gi|30794512|ref|NP_038579.1| hydroxymethylbilane synthase |  | Fwd:CAGGAATTCAGTGCCATCG  Rev:TATCCTGGTCCTTGGCTCG | 1426475_at  1436930_x_at |
| HPRT | Hprt1 | >gi|7305155|ref|NP_038584.1| hypoxanthine guanine phosphoribosyl transferase 1 |  | Fwd:GTACTTCAGGGATTTGAATCACG  Rev:AGATGGCCACAGGACTAGA | 1448736_a_at |
| LAP1B | Tor1aip1 | >gi|21450141|ref|NP_659040.1| lamina-associated polypeptide 1B |  |  | 1426084_a_at |
| LAP2 | Tmpo | >gi|6755817|ref|NP_035735.1| thymopoietin |  | Fwd:GTTCTCATCTAAGTACGTCCCGA  Rev:GGTTTCCATAGCTTGATAGACCA | 1452036_a_at  1421237_at  1426020_at  1426349_s_at  1428976_at |
| Lbr | Lbr | >gi|19527034|ref|NP_598576.1| lamin B receptor |  | Fwd:GAGGTTGGATAGTTTCCCAGTCA  Rev:GTGGTATGTGTAATAGAAACCCAAAT | 1415829_at |
| Lamin A | Lmna | >gi|50355692|ref|NP_001002011.1| lamin A isoform A |  | Fwd:GCACCGCTCTCATCAACT  Rev:TGAGCGCAGGTTGTACT | 1421654_a_at  1425472_a_at  1426868_x_at  1452212_at  1457670_s_at |
| Lamin C | Lmna | >gi|9506843|ref|NP_062263.1| lamin A isoform C2 |  | Fwd:GCACCGCTCTCATCAACT  Rev:TAGGCTGGCAGGGCTAC | 1421654_a_at  1425472_a_at  1426868_x_at  1452212_at  1457670_s_at |
| Lamin B1 | Lmnb1 | >gi|6754556|ref|NP_034851.1| lamin B1 |  | Fwd:ATGGAGATCAGCGCCTAC  Rev:CCACATCAACTCTCTTCCG | 1423520_at  1423521_at  1444459_at |
| Lamin B2 | Lmnb2 | >gi|6754558|ref|NP_034852.1| lamin B2 |  | Fwd:CGAGGAGGAGAGGCTGAA  Rev:GAGGTGTCCTCAGTCTCCA | 1451849_a_at  1448531_at |
| LUMA | Tmem43 | >gi|21311891|ref|NP_083042.1| transmembrane protein 43 |  |  | 1426434_at  1439418_x_at |
| Man1 | lemd3 | >gi|83028344|ref|XP_354566.4| PREDICTED: LEM domain containing 3 isoform 1 |  | Fwd:GGGAGCACTACTCGGACT  Rev:GCCGCCAGATTTAGCAG | 1459700_at  1435291_at |
| MEF2C | Mef2c | >gi|13384624|ref|NP_079558.1| myocyte enhancer factor 2C |  | Fwd:CAGCAGCAGCACCTACATA  Rev:GTGATCCTCTCGGTCGC | 1421027_a_at  1421028_a_at  1424852_at  1439946_at  1445420_at  1446484_at  1451506_at  1451507_at |
| Myogenin | Myog | >gi|13654247|ref|NP_112466.1| myogenin |  | Fwd:CTGCCCTGAGTTGAGAGAGAA  Rev:GAGACCTTGGTCAGACGG | 1419391_at |
| NET1 | Prpf38b | >gi|30794464|ref|NP_080121.1| PRP38 pre-mRNA processing factor 38 (yeast) domain containing B | gnl|CDD|17330 pfam03371, PRP38, PRP38 family |  | 1428674_at  1452869_at  1455016_at  1456506_at |
| NET2 | Sfrs11 | >gi|33469007|ref|NP_081265.1| splicing factor, arginine/serine-rich 11 |  |  | 1427269_at  1430077_at  1439095_at  1452371_at |
| NET3 (1) | Tmem48/NDC1 | >gi|28077005|ref|NP_082631.1| transmembrane protein 48 |  | Fwd:GTGTCTGCAATTTCTAACCCAT  Rev:AAAGTACATTATCAGCACCCG | 1460353_at  1424173_at  1433813_at |
| NET4 | Tmem53 | >gi|21312110|ref|NP_081113.1| transmembrane protein 53 | gnl|CDD|26417 pfam05705, DUF829, Eukaryotic protein of unknown function (DUF829) | Fwd:GTGGGCACCATCTTTGACA  Rev:AGCAAGCAGGAAGTGAAAC | 1451479_a_at |
| NET5 | D4Ertd429e | >gi|68342032|ref|NP_001020277.1| hypothetical protein LOC230917 isoform a |  |  | 1454868_at  1438529_at  1438010_at |
| NET6 | Nol9 | >gi|21311971|ref|NP_083003.1| hypothetical protein LOC74035 | gnl|CDD|26916 pfam06807, Clp1, Pre-mRNA cleavage complex II protein Clp1;  gnl|CDD|11057 COG1341, COG1341, Predicted GTPase or GTP-binding protein |  | 1429338_a_at  1432218_a_at  1432219_at  1453114_at  1455330_at |
| NET7/15 (2) | Noc2l | >gi|23956106|ref|NP_067278.1| nucleolar complex associated 2 homolog | gnl|CDD|15568 pfam03715, UPF0120, Uncharacterised protein family (UPF0120) | Fwd:GATGGAGACACAGACACGG  Rev:TCTGCTGTCCTCTCGCC | 1424323_at  1436136_at |
| NET8 | Lpgat1 | >gi|26986567|ref|NP_758470.1| lysophosphatidylglycerol acyltransferase 1 | gnl|CDD|24304 smart00563, PlsC, Phosphate acyltransferases;  gnl|CDD|25778 pfam01553, Acyltransferase, Acyltransferase | Fwd:CCGGTTGTCGCTCAGAT  Rev:GCTCCTGTAATTATGTTCAAGGTG | 1424349_a_at  1424350_s_at |
| NET9 | Tor1aip2 | >gi|46048324|ref|NP_766431.2| torsin A interacting protein 2 | gnl|CDD|14393 COG5271, MDN1, AAA ATPase containing von Willebrand factor type A (vWA) domain | Fwd:CAGAGAAGTTTGAAAGCCATTGAT  Rev:CTGCCCTACACCCTCCA | 1435526_at |
| NET10 | AK122209 | >gi|71274162|ref|NP_001025047.1| hypothetical protein LOC382038 |  |  | 1435379_at |
| NET11 | Sccpdh | >gi|30520019|ref|NP_848768.1| saccharopine dehydrogenase (putative) | gnl|CDD|26077 pfam03435, Saccharop_dh, Saccharopine dehydrogenase;  gnl|CDD|12604 COG3268, COG3268, Uncharacterized conserved protein |  | 1426510_at  1438909_at |
| NET12 | Wdr43 | >gi|82994960|ref|XP_484662.2| PREDICTED: WD repeat domain 43 isoform 1 | gnl|CDD|29257 cd00200, WD40, WD40 domain |  | 1428389_s_at  1428390_at |
| NET13 | 4122402O22Rik | >gi|27229251|ref|NP_084221.1| hypothetical protein LOC77626 |  |  | 1429530_a_at  1429531_at  1437024_at  1453182_a_at |
| NET14a (3) | Wdr33, isoform | >gi|18043557|gb|AAH19463.1| Wdr33 protein | gnl|CDD|29257 cd00200, WD40, WD40 domain | Fwd:TCACTGTTAAGGCAACTCCC  Rev:GCATCTCATCCAAAGGCTTC | 1453554_a_at  1423874_at  1455281_at |
| NET14b (3) | Wdr33 | >gi|21362285|ref|NP_083142.2| WD repeat domain 33 | gnl|CDD|29257 cd00200, WD40, WD40 domain | Fwd:CATGGGAAGGCCGCAGA  Rev:GTCATCAGGACCAGGAAAGT | 1418338_at  1444434_at  1444558_at  1453322_at  1456488_at |
| NET16 | Wdr75 | >gi|83523742|ref|NP_082875.1| WD repeat domain 75 | gnl|CDD|29257 cd00200, WD40, WD40 domain |  | 1442296_at  1451649_a_at |
| NET17 | BC027231 | >gi|22122411|ref|NP_666084.1| hypothetical protein LOC212547 |  |  | 1451439_at  1455781_at |
| NET18 | BC013481 | >gi|30425362|ref|NP_848541.1| hypothetical protein LOC245945 | gnl|CDD|25314 smart00360, RRM, RNA recognition motif |  | 1423411_at  1423412_at  1438021_at |
| NET19 (4)(5) | record removed |  |  |  |  |
| NET20 | BC052328 | >gi|38142474|ref|NP_938043.1| hypothetical protein LOC223433 |  |  | 1435375_at  1440718_at |
| NET21 | Utp15 | >gi|30725776|ref|NP_849249.1| Src-associated protein SAW | gnl|CDD|29257 cd00200, WD40, WD40 domain |  | 1434125_at  1454846_at |
| NET22 | Jmjd1b | >gi|54611251|gb|AAH38376.1| Jmjd1b protein | gnl|CDD|25904 pfam02373, JmjC, jmjC domain |  | 1428320_at |
| NET23 | 2610307O08Rik | >gi|38083636|ref|XP_128954.2| PREDICTED: hypothetical protein LOC72512 isoform 1 |  |  | 1427911_at  1447621_s_at |
| NET24 | 1200007D18Rik | >gi|13385678|ref|NP_080446.1| hypothetical protein LOC67458 |  |  | 1423333_at  1423334_at  1437908_a_at  1438792_at  1438793_x_at |
| NET25 | Lemd2 | >gi|22122563|ref|NP_666187.1| LEM domain containing 2 |  | Fwd:GCTAGACAGCACGGTGA  Rev:GCTGCTCCTACCTTCTATAACATC | 1424326_at |
| NET26 | Tmem14c | >gi|13384766|ref|NP_079663.1| transmembrane protein 14C | gnl|CDD|7127 pfam03647, UPF0136, Uncharacterized protein family (UPF0136) | Fwd:TCTCCAGACCTCCACATTACAA  Rev:AGCGTGAGCTGAAGTCG | 1416479_a_at |
| NET27 | D11Bwg0280e | >gi|54607102|ref|NP_001005867.1| hypothetical protein LOC52915 isoform 2 | gnl|CDD|17238 pfam02891, zf-MIZ, MIZ zinc finger |  | 1460739_at |
| NET28 | 1110015K06Rik | >gi|39930423|ref|NP_081024.1| hypothetical protein LOC68510 |  |  | 1426943_at  1443793_x_at |
| NET29 | 2010310D06Rik | >gi|29789387|ref|NP_766129.1| transmembrane protein induced by tumor necrosis factor alpha |  | Fwd:GCAACTCTTCAATGCGCT  Rew:AGTCCTTCTTGTTCCCGT | 1435031_at |
| NET30 | Mospd3 | >gi|28077073|ref|NP_084313.1| motile sperm domain containing 3 | gnl|CDD|25573 pfam00635, Motile_Sperm, MSP (Major sperm protein) domain |  | 1453961_a_at  1460452_at |
| NET31 | 2700094F01Rik | >gi|40254356|ref|NP_848740.3| hypothetical protein LOC72649 |  | Fwd:GCACTTCGTTCAGACGC  Rev:CGTATGAAACATATTATTCCTGCCC | 1426797_at  1452199_at |
| NET32 | Spfh2 | >gi|23956396|ref|NP_705820.1| | gnl|CDD|24227 smart00244, PHB, prohibitin homologues;  gnl|CDD|25697 pfam01145, Band_7, SPFH domain / Band 7 family | Fwd:CGTGTGTCTTGTGAAAGTGAGATA  Rev:ATAAGGAAACAAACAGTGGCTAGAA | 1435223_at |
| NET33 | Scara5 | >gi|22296589|ref|NP_083179.1| hypothetical protein LOC71145 | gnl|CDD|178 smart00202, SR, Scavenger receptor Cys-rich |  | 1431336_at  1451204_at |
| NET34 | Slc39a14 | >gi|47059049|ref|NP_659057.2| solute carrier family 39 (zinc transporter), member 14 | gnl|CDD|25919 pfam02535, Zip, ZIP Zinc transporter |  | 1425649_at  1427035_at  1438490_at  1457770_at |
| NET35 | 2610301G19Rik | >gi|31560870|ref|NP_666167.2| hypothetical protein MGC7730 |  |  | 1426012_a_at |
| NET36 | Tmem74 | >gi|30425256|ref|NP_780711.1| hypothetical protein LOC239408 |  |  | 1439807_at  1456684_at |
| NET37 | AI464131 | >gi|28495681|ref|XP_283952.1| PREDICTED: hypothetical protein LOC329828 isoform 1 | gnl|CDD|25679 pfam01055, Glyco_hydro_31, Glycosyl hydrolases family 31 | Fwd:GCACATGAATGACATTACACACT  Rev:CTCCACCTTCTCTGGCAA | 1435417_at |
| NET38 | Alg2 | >gi|31560366|ref|NP_064382.2| alpha-1,3-mannosyltransferase ALG2 | gnl|CDD|7651 pfam00534, Glycos_transf_1, Glycosyl transferases group 1;  gnl|CDD|10312 COG0438, RfaG, Glycosyltransferase |  | 1421059_a_at  1433144_at |
| NET39 | Ppapdc3 | >gi|21704026|ref|NP_663496.1| phosphatidic acid phosphatase type 2 domain containing 3 | gnl|CDD|8 smart00014, acidPPc, Acid phosphatase homologues;  gnl|CDD|24564 pfam01569, PAP2, PAP2 superfamily | Fwd:CCCTGGCCCACTAGATAC  Rev:AGAGAAGGCTCCTATGGTCA | 1424362_at  1442592_at |
| NET40 | A930041I02Rik | >gi|86198339|ref|NP_848893.2| hypothetical protein LOC320271 |  |  | 1440846_at  1445081_at |
| NET41 | Tysnd1 | >gi|20860640|ref|XP_125636.1| PREDICTED: trypsin domain containing 1 isoform 1 |  |  | 1428689_at  1428690_at  1441856_x_at |
| NET42 | Vwa2 | >gi|42741661|ref|NP_766428.2| A-domain containing protein similar to matrilin and collagen | gnl|CDD|29245 cd01472, vWA_collagen, von Willebrand factor (vWF) type A domain;  gnl|CDD|29248 cd01475, vWA_Matrilin, VWA_Matrilin |  | 1438567_at |
| NET43 | AI429152 | >gi|23346561|ref|NP_694766.1| expressed sequence AI429152 | gnl|CDD|16422 pfam05127, DUF699, Putative ATPase (DUF699);  gnl|CDD|11158 COG1444, COG1444, Predicted P-loop ATPase fused to an acetyltransferase |  | 1433692_at  1447601_x_at |
| NET44 | Slc25a22 | >gi|21311845|ref|NP_080922.1| solute carrier family 25 (mitochondrial carrier, glutamate), member 22 | gnl|CDD|25417 pfam00153, Mito_carr, Mitochondrial carrier protein |  | 1452653_at |
| NET45 | Dak | >gi|21703976|ref|NP_663471.1| hypothetical protein LOC225913 | gnl|CDD|25947 pfam02733, Dak1, Dak1 domain;  gnl|CDD|25948 pfam02734, Dak2, DAK2 domain |  | 1425300_at  1447244_at |
| NET46 (6) |  | >gi|27734797|ref|NP_775857.1| hypothetical protein LOC283238 [Homo sapiens] | gnl|CDD|25388 pfam00083, Sugar_tr, Sugar (and other) transporter;  gnl|CDD|12168 COG2814, AraJ, Arabinose efflux permease |  |  |
| NET47 | Tm7sf2 | >gi|58037315|ref|NP_082730.1| transmembrane 7 superfamily member 2 | gnl|CDD|16915 pfam01222, ERG4_ERG24, Ergosterol biosynthesis ERG4/ERG24 family |  | 1460684_at |
| NET48 | 4933424B01Rik | >gi|20270295|ref|NP_620096.1| hypothetical protein LOC71177 |  |  | 1419211_s_at  1429811_at  1455076_a_at |
| NET49 | Noc4l | >gi|31981994|ref|NP_705798.2| nucleolar complex associated 4 homolog | gnl|CDD|8841 pfam03914, CBF, CBF/Mak21 family;  gnl|CDD|14677 COG5593, COG5593, Nucleic-acid-binding protein possibly involved in ribosomal biogenesis | Fwd:ACTTGGCTGACCTCTTCC  Rev:CAGGCAGGGTGTCTACG | 1423826_at  1423827_s_at  1443794_x_at  1438095_x_at |
| NET50 | LOC209183 | >gi|82942779|ref|XP_127048.3| |  |  | none |
| NET51 | 0610007P14Rik | >gi|10946822|ref|NP_067421.1| hypothetical protein LOC58520 | gnl|CDD|5736 pfam03694, Erg28, Erg28 like protein | Fwd:CTGTGGACATTCCTCCTCG  Rev:TTTCTTCTGTCTGGATACTGGTT | 1454161_s_at  1458286_at |
| NET52 | 2600011C06Rik | >gi|82942735|ref|XP_905075.1| PREDICTED: RNA binding motif protein 25 isoform 14 | gnl|CDD|25309 smart00311, PWI, PWI, domain in splicing factors;  gnl|CDD|25314 smart00360, RRM, RNA recognition motif |  | 1425522_at  1425523_at  1425524_at  1428907_at  1428908_at  1437862_at  1444496_at |
| NET53 | 4831426I19Rik | >gi|27369682|ref|NP_766088.1| hypothetical protein LOC212073 | gnl|CDD|10914 COG1196, Smc, Chromosome segregation ATPases | Fwd:TTATGCCCAAGGCTGGAC  Rev:AAGGAGTGTGTAATCCCATCT | 1440402_at |
| NET54 | Tdrd9 | >gi|82943193|ref|XP_127120.5| PREDICTED: tudor domain containing 9 | gnl|CDD|28960 cd00079, HELICc, Helicase superfamily c-terminal domain;  gnl|CDD|24255 smart00333, TUDOR, Tudor domain  gnl|CDD|26244 pfam04408, HA2, Helicase associated domain;  gnl|CDD|11354 COG1643, HrpA, HrpA-like helicases |  | 1453357_at |
| NET55 | Aph1c | >gi|21492616|ref|NP_080950.1| anterior pharynx defective 1c homolog | gnl|CDD|26475 pfam06105, Aph-1, Aph-1 protein |  | 1429466_s_at |
| NET56 | Dullard | >gi|23956160|ref|NP_080293.1| Dullard homolog | gnl|CDD|3945 smart00577, CPDc, catalytic domain of ctd-like phosphatases;  gnl|CDD|14314 COG5190, FCP1, TFIIF-interacting CTD phosphatases, including NLI-interacting factor | Fwd:GGGCTTACAGGAGCCAC  Rev:GCTGTCACCAGAGCCTAT | 1452100_at |
| NET57 | D11Ertd759e | >gi|82935492|ref|XP_900130.1| PREDICTED: similar to chromosome 17 open reading frame 27 | gnl|CDD|29102 cd00162, RING, RING-finger (Really Interesting New Gene) domain;  gnl|CDD|28893 cd00009, AAA, AAA-superfamily of ATPases |  | 1455500_at  1460018_at |
| NET58 (4) | record removed |  |  |  |  |
| NET59 | Ncln | >gi|33469043|ref|NP_598770.1| nicalin homolog | gnl|CDD|17477 pfam04389, Peptidase_M28, Peptidase family M28;  gnl|CDD|26387 pfam05450, Nicastrin;  gnl|CDD|11940 COG2234, Iap, Predicted aminopeptidases |  | 1424203_at |
| NET60 | 2610304G08Rik | >gi|34328077|ref|NP_081710.1| hypothetical protein LOC70470 | gnl|CDD|15027 smart00582, RPR, domain present in proteins, which are involved in regulation of nuclear pre-mRNA |  | 1427887_at  1441005_at  1452581_at |
| NET61 | LOC623808 | >gi|82973371|ref|XP_128377.6| PREDICTED: similar to Nucleolar preribosomal-associated protein 1 |  |  | 1453999_at  1454841_at |
| NET62 | BC025519 | >gi|71725343|ref|NP_001025185.1| hypothetical protein LOC223722 | gnl|CDD|25592 pfam00698, Acyl_transf_1, Acyl transferase domain;  gnl|CDD|10205 COG0331, FabD, (acyl-carrier-protein) S-malonyltransferase;  gnl|CDD|12655 COG3321, COG3321, Polyketide synthase modules and related proteins |  | 1452216_at |
| NET63 (4) | record removed |  |  |  |  |
| NET64 | A330008L17Rik | >gi|30425226|ref|NP_780688.1| hypothetical protein LOC234624 |  |  | 1439870_at  1457318_at |
| NET65 (4) | record removed |  |  |  |  |
| NET66 (4) | record removed |  |  |  |  |
| NET67 | Crim2 | >gi|37590537|gb|AAH59909.1| Crim2 protein | gnl|CDD|24219 smart00216, VWD, von Willebrand factor (vWF) type D domain |  | 1440955_at |
| Ppia | Ppia | >gi|6679439|ref|NP_032933.1| peptidylprolyl isomerase A |  | Fwd:ATGAGAACTTCATCCTAAAGCATAC  Rev:CTTCCACAATGTTCATGCCT | 1417451_a_at |
| Rps18 | Rps18 | >gi|6755368|ref|NP_035426.1| ribosomal protein S18 |  | Fwd:TCTAGACAACAAGCTGCGT  Rev:AGGCCCAGAGACTCATT | 1435712_a_at  1448739_x_at  1455572_x_at |

Notes:

1. NET3 is the mouse homolgue of yeast NDC1, a novel nucleoporin.

2. NET7 and NET15 are the same protein.

3. NET14a and NET14b are alternative splice products.

4. The original record was moved from GenBank due to an update of the mouse genome build.

5. NET19 is suggested not to be a NE-enriched protein, as determined by immunoblotting of subcellular fractions with anti-peptide antibodies (E. Schirmer and L. Gerace unpublished data).

6. The mouse homologue of NET46 has not yet been identified.

7. The protein sequences were blast-searched against Rfam, Smart and NCBI COG databases. Listed are the returned “conserved protein domains” with an E value less than 6x 10^-3^.
