# Supplementary figures and images for: Nuclear envelope transmembrane proteins (NETs) that are up-regulated during myogenesis
Source: BMC Cell Biol. 2006 Oct 24;7:38. doi: 10.1186/1471-2121-7-38 (PMC1635557; doi:10.1186/1471-2121-7-38)

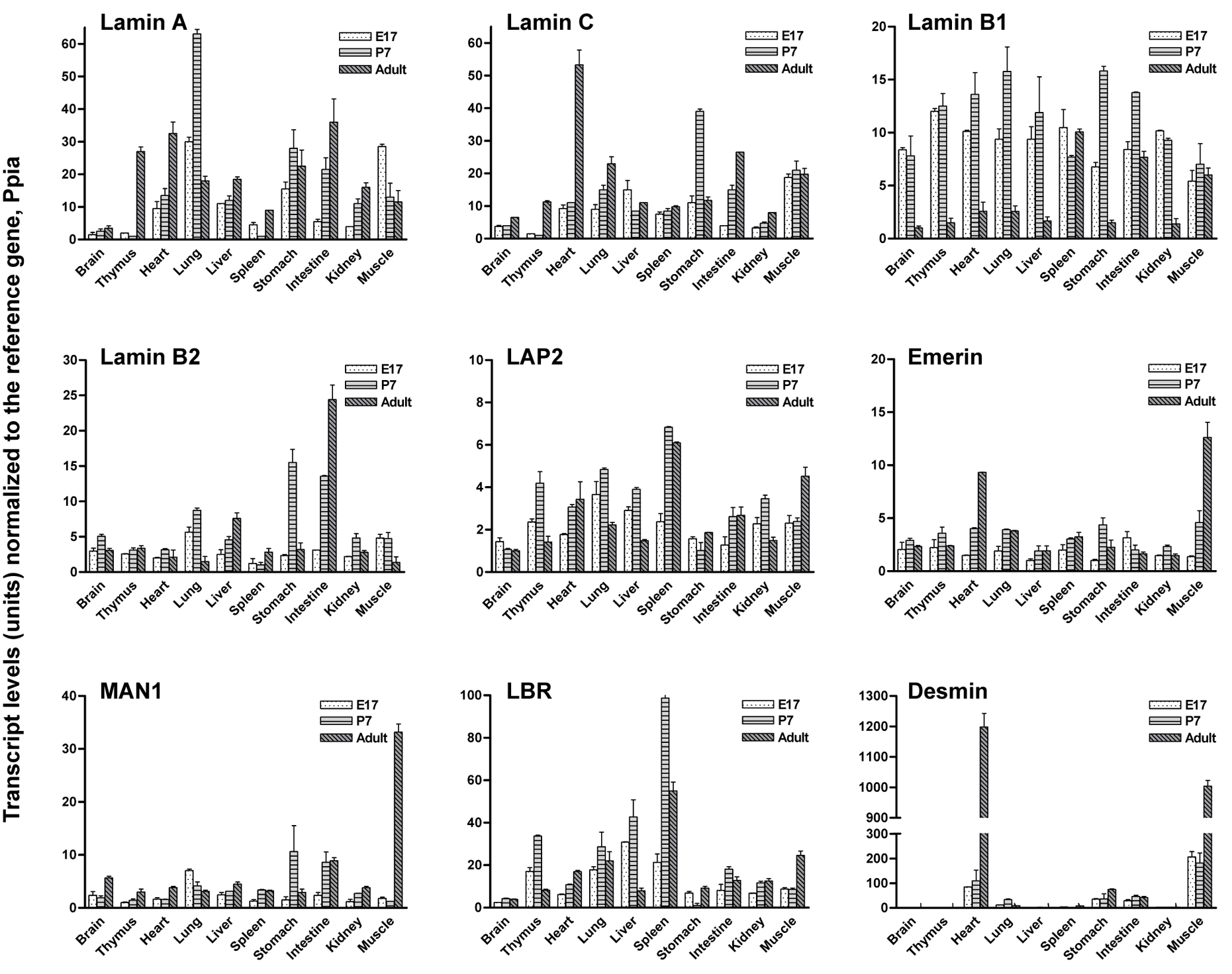

Supplement: Additional File 2 — Relative levels of NE markers and desmin at three developmental stages in 10 mouse tissues. Gene expression levels of selected lamina proteins were measured by quantitative real-time PCR in ten C129 mouse tissues from embryonic day 17 (E7), post-natal day 7 (P7) and adult. All signal values were normalized to peptidylprolyl isomerase A (Ppia) as in Fig. 4. The Y-axis indicates the signal value of RNA transcript after normalization to Ppia. The error bars indicate the standard deviation. [file 1471-2121-7-38-S2.tiff]

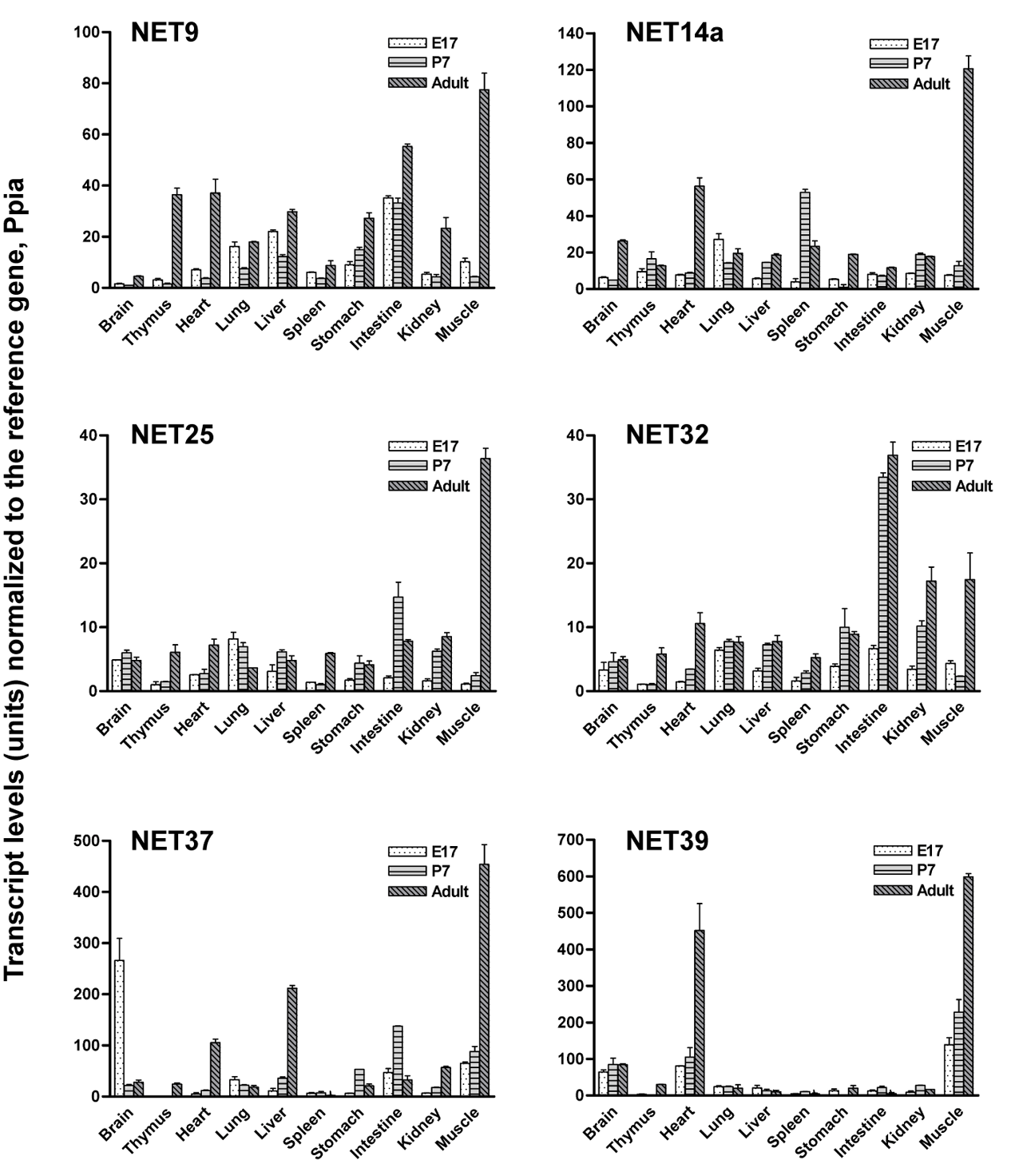

Supplement: Additional File 3 — Relative levels of NETs up-regulated in C2C12 differentiation at three developmental stages in 10 mouse tissues. Gene expression levels of the 6 NETs up-regulated during C2C12 differentiation were measured by quantitative real-time PCR in ten C129 mouse tissues from embryonic day 17 (E7), post-natal day 7 (P7) and adult. All signal values were normalized to peptidylprolyl isomerase A (Ppia) as in Fig. 4. The Y-axis indicates the signal value of RNA transcript after normalization to Ppia. The error bars indicate the standard deviation. [file 1471-2121-7-38-S3.tiff]

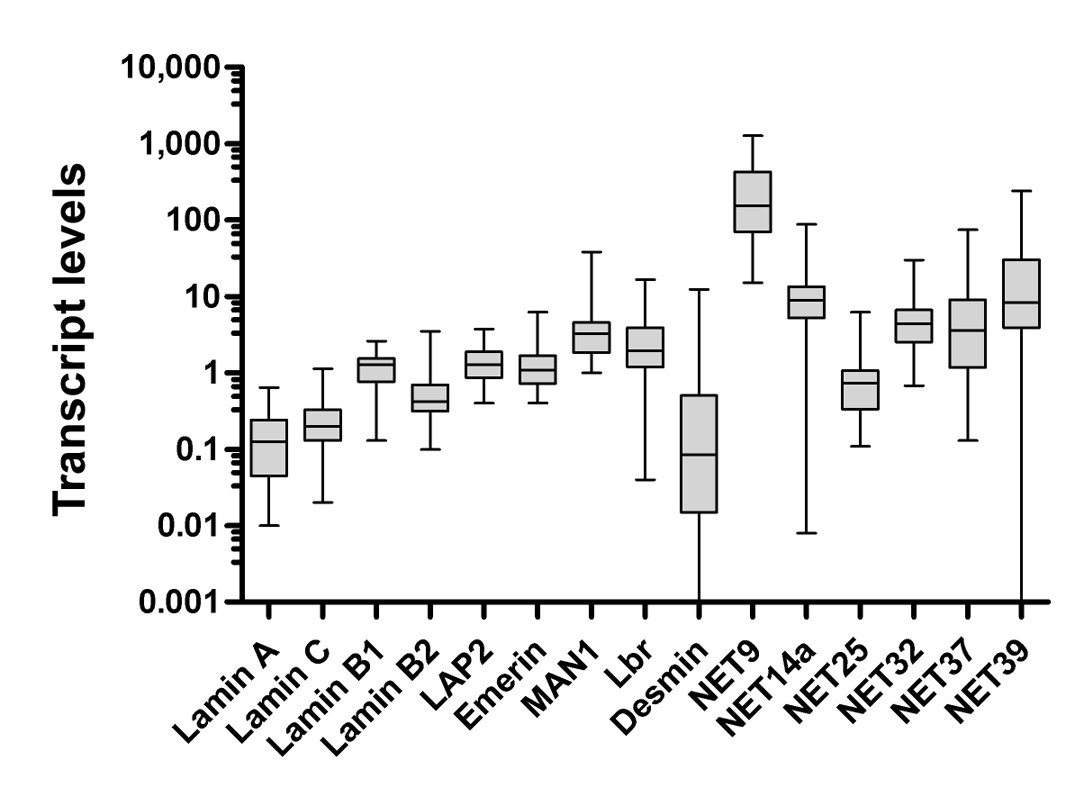

Supplement: Additional File 4 — Expression range of selected lamina proteins and the up-regulated NETs. A box-and-whisker graph shows gene expression levels of selected lamina proteins and the NETs in all tested mouse tissues. The expression values measured by RT-PCR in Additional File 2 and Additional File 3 were plotted on a log base 10 scale. Boxes indicate the median, the lower quartile and upper quartile values. Whiskers indicate the range of data points from the highest to the lowest. See [63]. [file 1471-2121-7-38-S4.tiff]
